# Metabolite profiling and network analysis reveal coordinated changes in grapevine water stress response

**DOI:** 10.1186/1471-2229-13-184

**Published:** 2013-11-20

**Authors:** Uri Hochberg, Asfaw Degu, David Toubiana, Tanya Gendler, Zoran Nikoloski, Shimon Rachmilevitch, Aaron Fait

**Affiliations:** 1 Albert Katz International School, Ben-Gurion University of the Negev, 84990 Sede Boqer, Israel; 2 the French Associates Institute for Agriculture and Biotechnology of Drylands (FAAB), the Jacob Blaustein Institutes for Desert Research, Ben-Gurion University of the Negev, 84990 Sede Boqer, Israel; 3 Max-Planck-Institut für Molekulare Pflanzenphysiologie, Am Mühlenberg 1, 14476 Potsdam, Golm, Germany

**Keywords:** Vitis vinifera, Grapevine, Metabolite profiling, Water deficit response, Stress physiology

## Abstract

**Background:**

Grapevine metabolism in response to water deficit was studied in two cultivars, Shiraz and Cabernet Sauvignon, which were shown to have different hydraulic behaviors (Hochberg et al. Physiol. Plant*.* 147:443–453, 2012).

**Results:**

Progressive water deficit was found to effect changes in leaf water potentials accompanied by metabolic changes. In both cultivars, but more intensively in Shiraz than Cabernet Sauvignon, water deficit caused a shift to higher osmolality and lower C/N ratios, the latter of which was also reflected in marked increases in amino acids, e.g., Pro, Val, Leu, Thr and Trp, reductions of most organic acids, and changes in the phenylpropanoid pathway. PCA analysis showed that changes in primary metabolism were mostly associated with water stress, while diversification of specialized metabolism was mostly linked to the cultivars. In the phloem sap, drought was characterized by higher ABA concentration and major changes in benzoate levels coinciding with lower stomatal conductance and suberinization of vascular bundles. Enhanced suberin biosynthesis in Shiraz was reflected by the higher abundance of sap hydroxybenzoate derivatives. Correlation-based network analysis revealed that compared to Cabernet Sauvignon, Shiraz had considerably larger and highly coordinated stress-related changes, reflected in its increased metabolic network connectivity under stress. Network analysis also highlighted the structural role of major stress related metabolites, e.g., Pro, quercetin and ascorbate, which drastically altered their connectedness in the Shiraz network under water deficit.

**Conclusions:**

Taken together, the results showed that *Vitis vinifera* cultivars possess a common metabolic response to water deficit. Central metabolism, and specifically N metabolism, plays a significant role in stress response in vine. At the cultivar level, Cabernet Sauvignon was characterized by milder metabolic perturbations, likely due to a tighter regulation of stomata upon stress induction. Network analysis was successfully implemented to characterize plant stress molecular response and to identify metabolites with a significant structural and biological role in vine stress response.

## Background

As one of the most widely cultivated fruit crops, grapes cover about seven million hectares of arable land worldwide (FAOSTAT, 2010). However, a large portion of the world’s wine producing areas are located in regions that currently suffer, or that are expected to encounter in the future, water deficits. In these areas, seasonal droughts coincide with the grapevine growing season (e.g., Mediterranean to semi-arid climates) [1], and the most important factor limiting grapevine growth in the Mediterranean is water stress [2]. In such areas, the combined effect of prolonged or recurrent drought events, large leaf-to-air vapor pressure gradients, and high air temperatures during the summer are known to limit grapevine yield, fruit metabolism and, consequently, wine quality [3-9].

Grapevines tend to adjust their leaf water balance by regulating the flow of water both to the leaf and from the leaf to the atmosphere. The plant’s hydraulic regulation is mediated by aquaporins [10] and vessel anatomy [11], and water loss via the leaves is regulated by stomatal conductance – which is, in turn, modulated by hormonal balance [12] – and leaf area [13]. It is generally accepted that *Vitis vinifera* cultivars possess significant variability in their hydraulic behavior [13], a feature reflected in the cultivar-specific responses to water deficit. Differences in drought tolerance between cultivars [12-15] are likely due to differences in root to shoot signaling and differential hydraulic regulation between cultivars [10,12]. Recently, quantitative trait loci (QTL) associated with grape hydraulic regulation were localized [16], suggesting a complex, regulatory process for this trait. The combination in grape cultivars of genotypic similarity and varied hydraulic behaviors established grapevines as an excellent model to study the molecular mechanisms underlying plant response to water deficit.

Plant molecular response to drought includes the production of compatible osmolytes [17]. Recent vine studies, including transcriptome and metabolome analyses, showed that processes associated with osmotic adjustment, protection against photoinhibition, and scavenging of reactive oxygen species were induced in response to drought conditions [18-22]. Water deficit also induces the synthesis of protective proteins, such as dehydrins and late-embryogenesis abundant (LEA) proteins, and the expression of water and ion transporters to maintain water and ion homeostasis [23,24]. In addition, prolonged stress can trigger changes at the leaf surface in the cuticle structure and cell walls can be triggered by prolonged stresses [25,26]. These mechanisms include the concerted action of large groups of genes [27-29]. Moreover, genotype-environment interaction further exacerbates the complexity of the stress response in grapevines [30-34].

In plants, Correlation Network (CN) has become an increasingly popular tool to represent the relationships of metabolites [35]. CN holds key features allowing for the analysis of coordinated changes of metabolites based on correlation coefficients. Moreover, CN enables the integration of information of diverse backgrounds (e.g. metabolites, genes, or physiological traits) elucidating the structure and regulation of a metabolic network, and it is employed in time-resolved experiments to identify genes regulating developmental and growth associated processes [36,37]. Metabolic CNs were employed, with genome-wide association mapping in *Arabidopsis* accession lines, to study the mode of inheritance of metabolic traits in seeds and fruits and their interactions [38]. Another study used CNs highlighting metabolic modules in a seed whose resilience to perturbation is indicative of the relevance of maintaining specific metabolite ratios [39].

In the present study, we explored the metabolic response of grapevine to progressive water deficit; changes in the central and specialized metabolisms of the two hydraulically different cultivars [40], Cabernet Sauvignon and Shiraz, were monitored during progressive water deficit treatment; leaf and sap metabolite profiles were integrated via network analysis to discern the metabolic basis of vine adjustments to stress. The findings are discussed with respect to the current knowledge about grape physiology and plant molecular responses to water deficit.

## Results

A significant variability was shown in the physiological responses to water deficit (D) between the cultivars Cabernet Sauvignon and Shiraz [40]. Here, the effect of progressive water deficit on leaf metabolism was investigated in the same plants. Leaves were analyzed by profiling their central and specialized metabolites, performing elemental analysis, and measuring osmolality.

**Figure 1:**
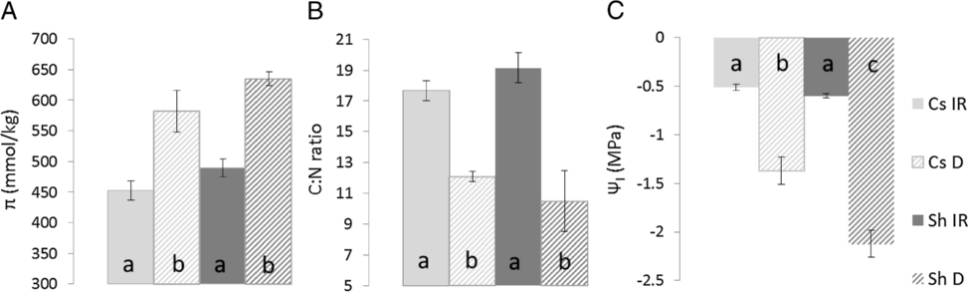
**Physiological adjustment in response to stress. (A)** Osmolality (π), **(B)** Carbon/Nitrogen ratio (C:N) and **(C)** leaf water potential (Ψ_l_) of Cabernet Sauvignon (Cs) and Shiraz (Sh) on day 34 of the experiment. Columns represent means ± SE (n = 6) and different letters represent significant difference between irrigated (IR) and water deficit (D) treatment as tested by the Student’s *t*-test (p-value < 0.05).

Periodic measurements of osmolyte content (Additional file 1: Table S1) suggested that significant changes in leaf metabolism occurred in both cultivars in response to progressive water deficit. Although osmolyte concentration (π) increased in D treated plants of both cultivars, the increase was sharper in Shiraz. For example, on day 34 of the experiment, π in Cabernet Sauvignon leaves was 582 mmol/kg whereas in those of Shiraz it was 635 mmol/kg (Figure 1). A high correlation was found between π and leaf water potential, Ψ_l,_ (R = 0.883, Additional file 2: Figure S1). Significantly lower C/N ratio (p < 0.05) was exhibited in plants subjected to D compared with plants exposed to the irrigated (IR) condition (Figure 1). Moreover, C/N differences between the treatments were larger in Shiraz (D was 45% lower) than in Cabernet Sauvignon (D was 32% lower).

### Comparative profiling of central and specialized metabolism of Shiraz and Cabernet Sauvignon under progressive water deficit

**Figure 2:**
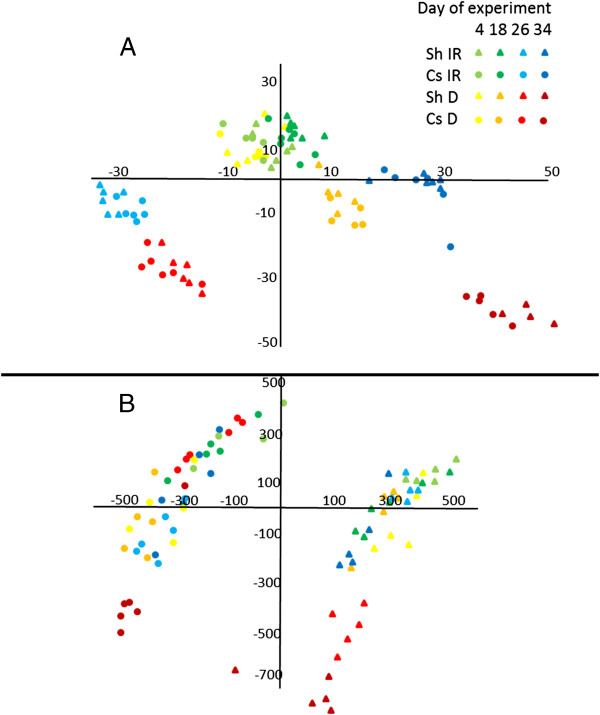
**Metabolic changes associated with water deficit and genotype.** Principle component analysis (PCA) plot (x – first component, y – second component) of Cabernet Sauvignon (Cs) and Shiraz (Sh) grape leaf extract of GC/MS based metabolites **(A)** and LC/MS based metabolite markers **(B)**. Symbols represent different sampling days and different cultivar treatments, i.e., irrigated (IR) and water deficit (D) treatments (n = 6).

**Figure 3:**
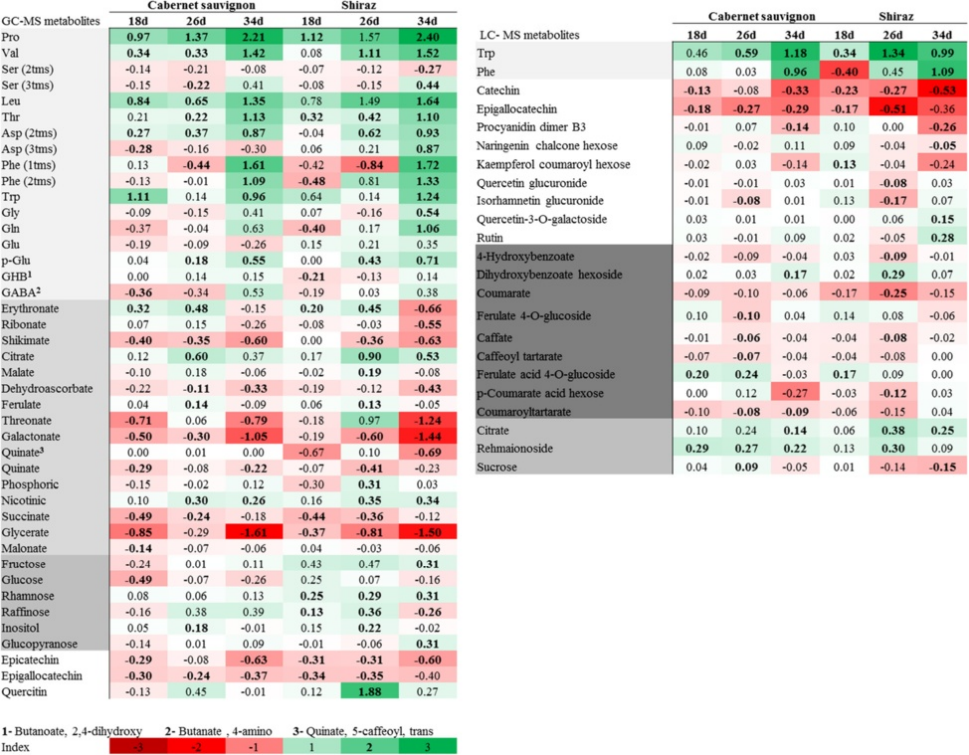
**Metabolic responses to progressive water deficit in leaves of Cabernet Sauvignon and Shiraz.** Values are the logarithmic transformed fold change (water deficit/irrigated) of selected leaf metabolites on days 18, 26, and 34 of the experiment. Bolded figures represent significant difference between irrigated and water deficit treatments as tested by the Student’s *t*-test (p-value < 0.05). Different colors represent the increase (green) or decrease (red) in metabolite logarithmic fold change as indicated in the color index (n = 6).

Metabolic profiling of the leaves unequivocally identified 69 annotated metabolites by GC/MS and 27 metabolites by LC/MS. Metabolite profiles of leaves were first analyzed by PCA (Figure 2). In the GC/MS based analysis of central metabolites (Figure 2A), the first principal component (PC1) – explaining the greatest variance (29%) across the dataset – separated the samples across sampling days (Figure 2A). Galactinol, Gly, quercetin, lignin precursors, ferulate and trans-5-*O*-caffeoyl-*D*-quinate [41] were the main metabolites contributing to the dispersion of the samples on PC1 (Additional file 1: Table S2). Levels of quercetin and trans-5-*O*-caffeoyl-*D*-quinate changed by up to 1000-fold (between the 26 and 34 days of experiment) during the course of the experiment (Additional file 3: Table S3). The second component (PC2) explained 28.3% of the variance and separated the samples according to the irrigated and water deficit treatments (Figure 2). Mainly changes in the abundance of Pro, galactinol, glycerate and galactonate (Additional file 1: Table S2) were responsible for sample dispersion along PC2. A significantly greater average fold change in metabolite abundance between the water deficit and irrigated treatments during later stages of the experiment (days 26 and 34) more clearly resolved the differences between Shiraz and Cabernet Sauvignon (Figure 3). Finally, the samples separated according to cultivar affiliation based on the third component (Additional file 2: Figure S2), which explained only 9% of the variance and was contributed mostly by quinate, quercetin and threonate suggesting an overall similarity in the grapevine metabolic response to progressive water deficit.

LC/MS based PCA (Figure 2B) showed that the two cultivars separated along PC1 (22.5% of the data variance), a finding attributed mainly to the tartaric esters (caffeoyl tartarate, caffeoyl tartarate dimer), 4-*O*-caffeoyl-*D*-quinate and quercetin-3-*O*-glucuronide. Expectedly, similar to the GC/MS based profiling, progressive water deficit led to increased separation between the treatments (Figure 2B). The LC/MS data subjected to Orthogonal Partial Least Squares Discriminate Analysis (OPLS-DA) identified metabolic markers for drought response (Additional file 2: Figure S3 and Additional file 2: Figure S4). OPLS-DA generated S-plot visualizing the magnitude of the contribution to the separation between water treatments made by molecular related ions (covariance) [42]. Accordingly, the larger effect of the treatment on the dataset was measured in Shiraz on day 34 of the experiment as reflected by the S-shaped data distribution (Additional file 2: Figure S3, Additional file 2: Figure S4). Among hundreds of markers, the analysis notably highlighted Trp, Phe, citrate, tartarate and catechin as highly affected by the D treatment in Shiraz (Additional file 2: Figure S3).

### Proline and branched chain amino acid accumulation correlate with water deficit driven changes in leaf water potential

GC/MS based metabolite profiling of leaves of the two vine genotypes showed larger changes in Shiraz than in Cabernet Sauvignon in response to progressive water deficit (Figure 3). In accordance with C:N ratio measurements (Figure 1), marked increases were observed in most amino acids (up to 251-fold) and a correspondingly strong decrease (down to 1/40) in the abundance of most organic acids in both cultivars in response to stress (Figure 3). On day 34 of the experiment, the amino acids Pro, Val, Leu, Thr and Trp were 251, 33, 43, 12 and 17 times higher, respectively, in water deficit than in irrigated samples in Shiraz. Similar results were found for Cabernet Sauvignon, where the same amino acids were elevated by 162, 26, 22, 13 and 9 times, respectively, in water deficit samples as compared with irrigated samples. Even Phe levels, of which initially decreased in plants under D treatment, was 52- and 21-fold higher on day 34 under water deficit conditions (Figure 3). Glu, the only amino acid to display opposite trends between the two cultivars in response to stress, increased by 1.5-fold in Shiraz but decreased by 1/2 in Cabernet Sauvignon across all sampling days (Figure 3).

Correlation analysis of the GC/MS based metabolite profile with physiological parameters showed that levels of Pro (R = 0.978), Val (R = 0.838) and Leu (R = 0.89) were strongly correlated with leaf water potential Ψ_l_ (Additional file 2: Figure S1B-D). Nonetheless, the contribution of amino acids to Ψ_l_ was relatively small. Comparison of amino acids, as was quantified by standard calibration curves, to the osmolyte concentration showed that amino acids accounted for less than 1% of π.

In response to stress, a decrease in most organic acids (with the exception of glycerate) was measured markedly in Shiraz and less in Cabernet Sauvignon (Figure 3). The most intensely depleted metabolites were glycerate and galactonate, which in the wake of water stress were reduced to 1/32 and 1/27 of their original levels, respectively, in Shiraz, and to 1/40 and 1/11 of their original levels, respectively, in Cabernet Sauvignon on day 34 of the experiment (Figure 3). In contrast, nicotinate were the only measured organic acid that accumulated in response to stress in both cultivars and at all three time points. Nicotinate was elevated by 1.44-, 2.2-, and 2.2-fold, at day 18, 26 and 34 respectively, in Shiraz, and by 1.25-, 1.99- and 1.81-fold, respectively, in Cabernet Sauvignon (Figure 3). Finally, inconsistent trends were observed for TCA intermediates during the experiment and between the cultivars. For example, like citrate, malate showed a significant accumulation in response to water deficit, but only in Shiraz (day 26). While succinate levels in both cultivars were significantly lower (1/3) in stressed plants on days 18 and 26 of the experiment, they showed no significant change on day 34 (Figure 3), likewise fumarate did not change across the experiment. Taken together, the extent of the changes in primary metabolism under severe stress (day 34) was greater in Shiraz as compared with Cabernet Sauvignon; both the average fold change of each metabolite and the number of significantly changed metabolites (Shiraz- 30, Cabernet Sauvignon- 18) were larger in Shiraz (Figure 3).

### Changes in specialized metabolism under progressive water deficit are genotype specific

Changes in the secondary metabolism were generally milder in magnitude than those measured for the central metabolites, and they were greater in Shiraz than in Cabernet Sauvignon (Figure 3). Furthermore, the OPLS-DA on the LC-MS dataset, which included hundreds of markers, highlighted mostly primary metabolites (citrate, tartarate, Phe and Trp) as major contributors to the differences between treatments (Additional file 2: Figure S3, Additional file 2: Figure S4). The flavanols catechin, epicatechin, epigallocatechin and procyanidin dimer B3 and the non-flavonoid phenolic compounds were significantly decreased under water deficit in both genotypes (Figure 3). In contrast, the abundance of quercetin-3-*O*-galactoside and rutin increased significantly (1.4- and 1.9-fold, respectively) in Shiraz water deficit plants but not in Cabernet Sauvignon (Figure 3).

### Correlation-based network analysis to identify coordinated stress induced metabolic perturbation

Four networks were generated by correlation-based network analysis of the four sets of data profiles from the two different cultivars (Shiraz and Cabernet Sauvignon) under the two conditions (D and IR) across the sampling data-points: Shiraz water deficit, Shiraz irrigated, Cabernet Sauvignon water deficit, and Cabernet Sauvignon irrigated plants (Additional file 1: Table S4). At q-value < 0.01 and r > 0.9, water stress caused a slight increase in the number of edges in Cabernet Sauvignon from 870 to 979, network density, from 0.3 to 0.32, and average node degree, from 22.6 to 24.78. Overall, Shiraz networks were characterized by greater numbers of edges compared to Cabernet Sauvignon networks. Under irrigated conditions the Shiraz network had 1352 edges, which markedly increased by 50% under water deficit conditions (Additional file 1: Table S4).

To investigate the statistical significance of network differences we performed permutations test (see Methods). Interestingly, none of the Cabernet Sauvignon IR permutations resulted in viable networks. Exclusively, all resulting networks contained zero nodes and consequently no edges. The 1000 permutation networks generated for the Cabernet Sauvignons D dataset, resulted in 406 no network, 359 one-node, 166 two-node, 52 three-node, 12 four-node, 4 five-node, and 1 six-node network. Due to the non-comparability of the permutation network parameters to the initially observed network parameter, the differences recorded between Cabernet Sauvignon IR and Cabernet Sauvignon D are highly significant (p < 0.001). A different picture arose for the comparison of the Shiraz permuted networks to the Shiraz initial networks. Here, all permutations resulted in viable networks for Shiraz IR permuted as well as for Shiraz D permuted. Nevertheless, none of the monitored differences in network parameters equaled or exceeded the initially observed difference between treatments, respectively, e.g., the difference of network density of Shiraz D (0.62) and Sh IR (0.45) resulting in a value of 0.17 was never achieved. These findings also render the differences between treatments highly significant (p < 0.001).

**Figure 4:**
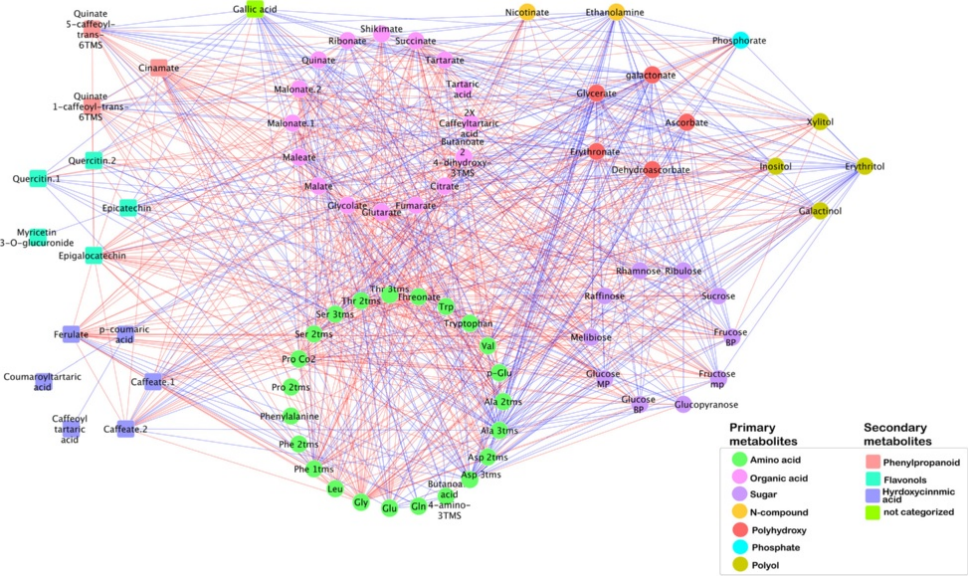
**Changes in Cabernet Sauvignon metabolite interactions as a result of water deficit.** Nodes correspond to primary (circles) and secondary (squares) metabolites; node colors correspond to compound classes as detailed in the figure legend. Edges between nodes represent correlations identified as significant at *r* ≥ 0.9 and q ≤ 0.01, where blue edges correspond to the irrigated treatment and red edges correspond to the water deficit treatment. Nodes are ordered into modules corresponding to their compound classes.

**Figure 5:**
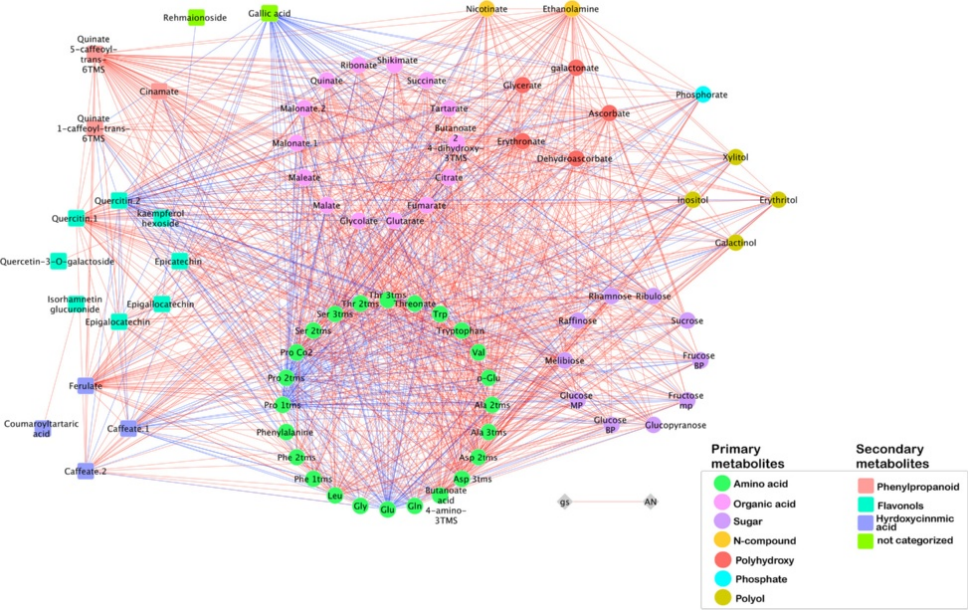
**Changes in Shiraz metabolite interactions as a result of water deficit.** Nodes correspond to primary (circles) and secondary (squares) metabolites; node colors correspond to compound classes as detailed in the figure legend. Edges between nodes represent correlations identified as significant at *r* ≥ 0.9 and q ≤ 0.01, where blue edges correspond to the irrigated treatment and red edges correspond to the water deficit treatment. Nodes are ordered into modules corresponding to their compound classes.

The varietal difference in metabolic response to stress can be appreciated by comparing the graphs (Figures 4, 5) where red edges are specific to the water deficit treatment. Between-cultivar differences are visualized in the symmetric difference networks (SDN, Additional file 2: Figure S6 and Additional file 2: Figure S7), in which blue edges are specific to Shiraz and red edges are specific to Cabernet Sauvignon. The SDN emphasize the presence of highly connected cultivar specific nodes. For example, the irrigated SDN comprises 77 nodes and 1120 edges, 319 edges are specific to Cabernet Sauvignon and 801 are specific to Shiraz, a ratio of 1 to 2.51 (Additional file 1: Table S4). Notably, all 40 edges of the lignin precursor trans-5-*O*-caffeoyl-*D*-quinate are specific to Cabernet Sauvignon, while ethanolamine has 36 edges, of which 31 are specific to Cabernet Sauvignon. In contrast, epigallocatechin has 47 edges, 46 of which are specific to Shiraz, and 43 of quercetin’s 44 edges are also specific to Shiraz. Similar results were found for the SDN created for the water deficit treatment. This network comprised 74 nodes and 1163 edges, of which 99 are specific to Cabernet Sauvignon and 1064 are specific to Shiraz, a ratio of 1 to 10.75. The predominance in the two SDNs of Shiraz specific edges, especially under progressive water deficit conditions, emphasizes the extent to which Shiraz underwent coordinated metabolic rearrangements. The nodal degrees of epigallocatechin (28 edges), quercetin (58 edges) and ethanolamine (52 edges) were particularly high in the Shiraz cultivar.

To estimate the extent of change in network structure during the progressive water deficit treatment and to identify metabolites with stress related structural features, the ratios of each metabolite nodal degree between the irrigated and water deficit networks were quantified (Additional file 1: Tables S5, Additional file 1: Tables S6). In both cultivars gallic acid played an important role in the structure of the network under irrigated conditions, but under water deficit conditions, the bulk of its contribution to connectivity was lost. Among the 15 metabolites in Shiraz most dramatically altered in terms of connectedness, 10 were amino acids, together with Ser-derived ethanolamine, ascorbate, fumarate, butanoate derivatives, ferulate and quercetin, and, some were sugars, including ribulose, melibiose and raffinose. Notably, the analysis highlighted the change in the structural role of Pro within the Shiraz network: under conditions of progressive water deficit, Pro lost all of its relations with the rest of the network, likely acquiring a unique role as an osmolyte. In Cabernet Sauvignon, among the first 15 metabolites whose connectedness was altered, six were amino acids. Of the organic acids, succinate, threonate, glutarate, malonate and 2,4 di-hydroxy benzoate exhibited changes in their connectedness. Among Cabernet Sauvignon sugars, raffinose was the most powerfully affected by the conditions of water deficit, but its nodal degree (1/3) was smaller in Cabernet Sauvignon than in Shiraz.

### Sap metabolism and stress response

**Figure 6:**
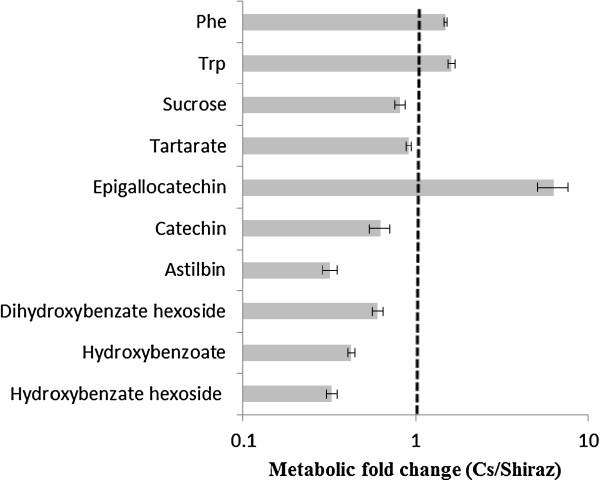
**Metabolites in the sap of Cabernet Sauvignon (Cs) and Shiraz in irrigated plots.** Values are the fold change Cs/Shiraz. Presented is the analysis of the leaves of irrigated plants sampled on day 4 of the experiment. Shown are metabolites that were significantly different (p-value < 0.05) between the cultivars in the irrigated plots on at least one of the sampling days and that showed similar trends throughout the experiment (Additional file 3: Table S3). Columns represent means ± SE (n = 6). The dashed line marks values of fold change equal to ‘one’, i.e., no change between relative metabolite contents of Shiraz and Cabernet Sauvignon cultivars.

**Figure 7:**
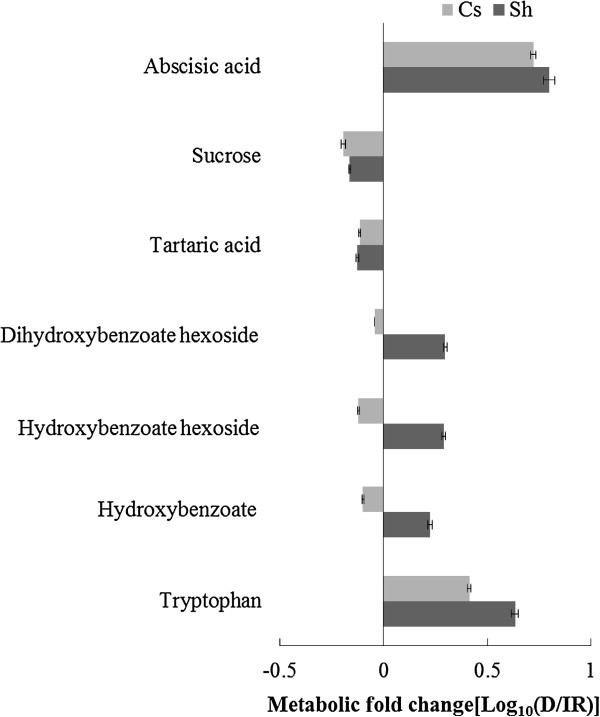
**Sap metabolic response of Cabernet Sauvignon (Cs) and Shiraz (Sh) to water stress.** Values are the logarithmic transformed fold change (water deficit/irrigated) of sap metabolites of Cabernet Sauvignon (Cs) and Shiraz on day 18 of the experiment. Only metabolites that were significantly different between irrigated and water deficit treatments as tested by the Student’s *t*-test (p-value < 0.05) are presented. Columns represent means ± SE (n = 6).

**Figure 8:**
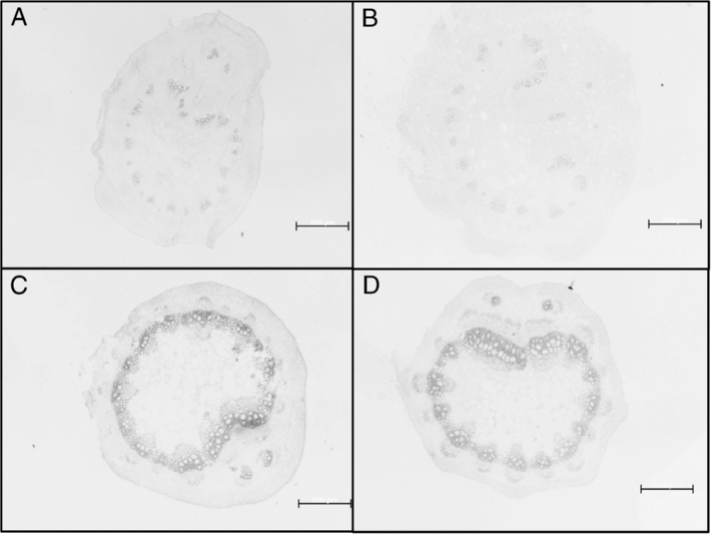
**Suberin accumulation in response to water deficit.** Cross section of petioles dyed with aniline blue for suberin (in dark grey) estimation for Cabernet Sauvignon (Cs) irrigated **(A)**, Shiraz irrigated **(B)**, Cabernet Sauvignon water deficit **(C)** and Shiraz water deficit **(D)** treatments on day 34 of the experiment. Bars = 500 μm (n = 6). Fluorescence reflectance (i.e., suberin accumulation) of the tissue from D plants **(C,D)** increased in Shiraz (3.21 fold) and in Cs (2.01 fold) compared to in the tissue from IR plants **(A,B)**.

LC/MS analysis of the phloem sap of plants grown under irrigated conditions identified 20 metabolites, 10 of which were significantly different between the cultivars (Figure 6). Compared to Cabernet Sauvignon, Shiraz had higher levels of astilbin (3-fold) and hydroxybenzoate (1.7 - 3-fold), whereas Cabernet Sauvignon sap was characterized by a markedly higher level of epigallocatechin (6.3-fold) than Shiraz (Figure 6). Levels of the coumarate derivatives coumaroyl tartarate and p-coumarate hexose varied significantly between the cultivars, but no consistent trend was observed during the experiment. Likewise, abscisic acid was identified in the sap of irrigated Cabernet Sauvignon at a higher level (1.71-fold) than in Shiraz, but this pattern was not consistent throughout the experiment (Additional file 2: Figure S3). Nevertheless, its content increased 5 - 6-fold in response to water deficit in both cultivars (Figure 7), and it was found to be strongly correlated with stomatal conductance g_s_ (R = -0.916, Additional file 2: Figure S5). Genotypic differences were shown for all three detected hydroxybenzoate forms (hydroxy benzoate, hydroxy benzoate hexoside and dihydroxy benzoate hexoside), which in response to water deficit accumulated in the sap of Shiraz (1.7-1.9-fold) but did not change in Cabernet Sauvignon (Figure 7). Hydroxybenzoate derived suberin was consistently shown to accumulate under water deficit conditions in the vascular systems of both cultivars. After 34 days without irrigation, suberin levels doubled in Cabernet Sauvignon and more than tripled in Shiraz, compared with the cultivars under the irrigated treatment (Figure 8).

## Discussion

Grapevine response to water deficit varies between cultivars apparently with respect to cultivar hydraulic behavior and genotype. Nevertheless, few studies have investigated the variability in the metabolic response to water deficit between genotypes. Here, two vine cultivars, Shiraz and Cabernet Sauvignon, with different hydraulic behaviors [40] were exposed to long-term (5 weeks) water deficit. Shiraz showed larger stress related changes in metabolite abundance and in the number of significantly altered metabolites. Across the metabolite profiles of central and secondary metabolism, primary metabolites were significantly more responsive to the stress compared to the identified secondary metabolites.

During the course of the experiment, the observed reduction in leaf water potential (Ψ_l_) could be partly explained by the increase in osmolality (π), which was found to be correlated with amino acid content. The stress-induced increase in amino acids (Figure 3) was previously observed in grapevines [20,43] and in other plant species [44]. The most significant increase in abundance among the amino acids was that of Pro, which was also strongly correlated with Ψ_l_ and contributed most of the variance to the dispersion along PC2 (in the PCA analysis, Figure 2A, Additional file 1: Table S2) which differentiate between the irrigation treatments. Pro accumulation is one of the most common and well-known responses of plants to dehydration [45-47]. However, when quantified against standard calibration curves, our measurements detected very small Pro concentrations, rendering it of negligible osmotic significance. This finding supports previous ones showing that compared to inorganic ions, amino acids made relatively small contributions to osmotic potential [48,49].

Processes that have been suggested to contribute to the accumulation in amino acids that leads to the observed decrease in the C:N ratio include the oxidative stress response [50], enhanced protein catabolism, nitrogen re-allocation driven by growth inhibition [51,52], and a shift in the proteome expressed as the production of greater numbers of stress-associated proteins [22,32], thereby enhancing, as our results imply, the biosynthesis of amino acids at the expense of C metabolites. Alternatively, the reduction in the C:N ratio measured in our study may be the result of the increased proportion of photorespiration/photosynthesis measured in grape leaves under stress conditions [40]. For example, it was shown that NO_3_ assimilation is dependent on photorespiration [53,54], and therefore, nitrogen assimilation is likely enhanced (proportionally to carbon) under stress conditions. That being said, other studies have suggested that the stimulation of N uptake and N assimilation are regulated by photosynthesis (for reviews see Lillo [55]) and that under drought stress the inactivation of nitrate reductase lowers N assimilation [56]. These lines of evidence emphasize the complexity of plant responses to stresses, dictating the need for additional comparative works into the regulation of C/N status and amino acid functional role under water stress.

The maintenance of higher leaf water potential by Cabernet Sauvignon could be attributed to a higher basal ABA level in the phloem sap leading to lower stomatal conductance under mild to moderate stress (g_s_ > 0.05 mol m^-2^ s^-1^), as defined by [57]. This hypothesis is further supported by the high correlation found between stomatal conductance and ABA and by previous findings of the involvement of ABA in the control of the former [12]. ABA is thought to control stomatal opening by affecting the biochemistry of guard cells, and by changing leaf hydraulic conductivity through modulation of permeability within vascular tissues, likely affecting aquaporin regulation [58-60]. Additionally to its role in hydraulic regulation, ABA is a central regulator of plant stress responses and was shown to modulate growth [60] and sugar transport, synthesis, and degradation [61,62]. Having said that, under severe stress (g_s_ < 0.05 mol m^-2^ s^-1^), sap ABA levels were similar in both genotypes. It is possible that cultivar variability in the above mentioned downstream processes of ABA contributed to the metabolic differences measured between the cultivars.

The link between stress tolerance and the regulation of water transport was suggested to be influenced by anatomical changes in the plant [63]. In the present study suberin was shown to accumulate under water deficit more in the vascular bundles of Shiraz than in those of Cabernet Sauvignon (Figure 8). Along with cutin, suberin is involved in the control of the movement of gases, water and solutes in plants [64]. While little is known about this fatty-acid–derived insoluble polymer, according to De Simone et al. [65], more than 85% of the aromatic moiety of suberin is composed of hydroxybenzoate [66]. Sap metabolite profiling identified increased abundance of benzoate and ferulate derivatives in the extracts of Shiraz when exposed to water deficit, suggesting enhanced suberin biosynthesis. Moreover, changes in the levels of metabolized shikimate, quinates, coumarines and flavonoids strongly suggest modulation of the phenylpropanoid pathway. Additionally, gallic acid and quinate, both linked with the phenylpropanoid pathway [67] and associated to ROS scavenging [68], were highlighted by the asymmetric networks to be highly coordinated under IR and D conditions respectively. Known as a general stress response in plants [67], the phenylpropanoid pathway can support generation of the building blocks for suberin [69]. Lastly, the association between suberin accumulation and differences in hydraulic behavior was previously observed in grapevines, lending support to our results [10]. Taken together, these lines of evidence suggest that enhanced suberin biosynthesis is induced in Shiraz to cope with the increasingly lower leaf water potential. Significant genotypic differences observed in quinate metabolism (1 caffeoyl quinate was abundant in Shiraz vs. trans-5-*O*-caffeoylquinate in Cabernet Sauvignon) and the existing gaps in the scientific knowledge about caffeoyl quinate metabolism dictate the need for more work.

Correlation-based network analysis (CNA) revealed that extensive topological network differences exist between the two grape cultivars. CNA of plants exposed to water stress showed a marked increase in the coordinated metabolic activities of the Shiraz cultivar in contrast to Cabernet Sauvignon plants, the latter of which were able to withstand the water stress with less metabolic changes. In addition, CNA emphasized the structural role of key stress metabolites, e.g., Pro. The increased network density and connectedness shown here, suggestive of tighter regulation imposed on metabolism, contradict the hypothesis that stress lowers the number of relations and subsequently has a negative effect on network stability [70]. To the best of our knowledge, an increase in metabolic network connectivity in response to water stress has not been observed in any organism. However, Sanchez et al. [71] showed that the correlation coefficient between metabolites of Lotus genotypes increased when they were subjected to salt stress, which may lead to higher network connectivity. We hypothesize that regulatory mechanisms under water deficit induce a concerted change in metabolism that allows the cell to cope with the new condition and that leads to more dependent metabolic profiles. The differential magnitudes (as shown by CNA) of the metabolic changes undergone by the two cultivars exposed to water deficit likely reflect corresponding differences in water stress tolerance. Cabernet Sauvignon, therefore, appears to be more stress tolerant than Shiraz, and as such, it does not require extensive, coordinated metabolic shifts in the wake of its exposure to stress.

Our analysis stresses the link between primary metabolism and water deficit, while variability in secondary metabolism was cultivar-dependent. The data suggest that *Vitis vinifera* cultivars possess qualitatively similar metabolic responses. The magnitude of each, at least in part, depends on the plant’s capacity to maintain its water balance. Metabolic data integrated via network analysis indicate that hydraulic regulation is a prominent modulatory element used to ameliorate the perturbation of cellular metabolism and highlights its benefits to the plant exposed to severe stress.

## Conclusions

*Vitis vinifera* cultivars undergo a generally conserved and highly coordinated metabolic shift during their stress responses. Changes in leaf metabolite content were evident based on the observed increase in osmolality and shift toward smaller C:N values. Both processes reflected the general accumulation of amino acids, a few of which, including Pro, were found to be correlated with Ψ_l_. The role of amino acid accumulation in the vine in response to water deficit, however, must be more thoroughly assessed. Grapevine sap analysis highlighted the association of ABA and benzoate metabolism with the stress response. Quantitative differences between grapevine cultivars revealed by network analysis but not yet fully understood, indicate the need for further research to elucidate the genotype specific details of physiological and molecular modulation. Network analysis was shown as an effective method to display differential response to stress among genotypes and identify biologically relevant metabolites.

## Methods

### Trial design

A greenhouse trial was established during May to June 2011 [40]. Two irrigation treatments were applied: water deficit, in which plants were irrigated to saturation only on day 0 and received no irrigation for the remainder of the experiment, and irrigated, in which plants were irrigated every four days to saturation. One-year-old *Vitis vinifera* L. cv. Shiraz and Cabernet Sauvignon, grafted on Richter 110, were planted in 9-L plastic pots, which were filled with 8 L of potting media (RAM8, Tuf Merom Golan, Israel) and covered with aluminum foil to reduce evaporation. The vines were trained on 2-m bamboo stakes and placed in a randomized complete block design. Throughout the experiment, daytime and nighttime greenhouse temperatures were kept between 26 ± 2.5 and 17 ± 1.5°C, respectively. Plants were moved randomly every four days throughout the greenhouse to avoid spatial effects. The trial continued until the Shiraz water deficit treatment plants began to wilt (Ψ_l_ reached a level of -2.12 MPa).

Leaves taken from six Cabernet Sauvignon and Shiraz vines, each under the water deficit or irrigated treatment, were sampled on each of the four sampling dates: days 4, 18, 26, and 34 of the experiment. The two irrigation treatments were identical until the fourth day of the experiment, when the irrigated plants received irrigation for the first time since sampling. Vines were tested for water potential (Ψ_l_), stomatal conductance (g_s_), net photosynthesis (A_N_), osmolality (π) and metabolite profile. On day 34, vine carbon/nitrogen ratios were also sampled. Due to the destructive nature of some of these methods, plants that were sampled were removed from the experiment. In addition, due to metabolome sensitivity, plants were not pruned at any time during the experiment.

### Leaf water potential (Ψ_l_)

Leaf water potential (Ψ_l_, MPa) was measured using a pressure bomb chamber (Arimad 3000, Israel) at midday. Measurements were taken from fully expanded, sun exposed, mature leaves (the same leaves that had been measured for photosynthetic parameters shortly beforehand). At each time point, six leaves per treatment (one leaf per plant) were selected. Immediately before excision, a plastic bag was placed over the leaf lamina. Each leaf was excised from the shoot using a scalpel blade and then placed into the pressure chamber with the petiole protruding from the chamber lid. The chamber was pressurized using a nitrogen tank, and Ψ_l_ was recorded as soon as xylem sap was observed emerging from the cut end of the petiole.

### Gas exchange measurements

Gas exchange measurements were conducted at midday according to Flexas et al. [72] on the youngest fully mature leaves. All measurements were carried out in the greenhouse. Measurements were conducted on days 4, 18, 26 and 34 of the experiment. A LiCor6400 portable photosynthesis system (Licor, Nebraska, USA) was used to measure stomatal conductance (mol H_2_O m^-2^ s^-1^) and net assimilation (μmol m^-2^ s^-1^). The leaves were exposed to a light intensity of 1000 PPFD and a CO_2_ concentration of 400 ppm while leaf temperature was kept at 25°C and relative humidity was between 30 and 55%.

### Osmolality (π)

For osmolyte concentration measurements, leaves from the greenhouse were macerated in liquid N_2_ and ground, after which 25 μg of the ground material was transferred to a 2-ml eppendorf tube and 50 μl of double distilled water was added. The eppendorfs were shaken for 10 min at 30°C, 1000 RPM and were then centrifuged or 4 min at 20,817 × g. After that, 10 μl of supernatant was used to determine the osmolality using a vapor pressure osmometer (Vapro® 5520, Wescor, USA). The value was then multiplied by the dilution factor of three.

### Elemental analyzer

Leaf samples were dried (60°C, for 96 h) and ground to powder. Samples of 2.7 mg were analyzed by a FlashEA™1112 CHNS-O Analyser (Thermo Fisher Scientific Inc., UK).

### Cross section and suberin staining

On day 34 of the experiment, petioles of the youngest fully mature leaves, from six plants of each cultivar and treatment were sampled and cross sectioned. The sections were fixed for 48 h in a 0.5:0.5:9 solution of formaldehyde, acetic acid and ethanol (70%), respectively. Tissue sections were dehydrated using a graded ethanol series (50, 70, 95, and 100%, 30 min each) followed by immersion in tert-butanol (8 h) and embedding in Paraplast Plus. After hardening, 8-μm thick cross sections were cut with a rotation microtome (RM2235, Leica, Nussloch, Germany). Cross sections were collected on glass slides and placed on a warming tray (40°C, 3 h). The tissue sections were de-paraffinized in xylene (33°C, 10 min) and rehydrated (ethanol 100, 95, 70, and 50%, 5 min each). The cross sections were stained with aniline blue and differences in the suberization were analyzed using fluorescence microscopy [73]. Images were processed and quantified using *ImageJ* software [74]. Observing only the blue channel, we ignored values lower than 30, which was found to be the background value. The expected value of each histogram was calculated and averaged.

### Sampling and extraction of leaves for metabolite profiling

Sampling, storage and extraction of the samples were done according to the recommended metabolite data reporting protocol [75]. At all sampling dates, leaf samples were collected, snap frozen immediately with liquid nitrogen and kept at -80°C until further analysis. Samples were extracted for parallel metabolite profiling (liquid and gas chromatography/mass spectrometry—LC/MS and GC/MS) as described in Weckwerth et al. [76]. Leaf tissue was grounded under liquid nitrogen using a RETCH-mill with pre-chilled holders and grinding beads. The frozen powder was weighed (70 mg), and metabolites were extracted in a 1 ml pre-chilled methanol:chloroform:water extraction solution (2.5:1:1 v/v). Internal standards, i.e., 0.2 mg/ml ribitol in water, 1 mg/ml ampicillin in water, 1 mg/ml corticosterone in methanol and 5 mg/ml heptadecanoic acid in chloroform, were subsequently added. The mixture was then briefly vortexed, centrifuged for 2 min at 20,817 × g (microcentrifuge 5417R), and the supernatant was decanted into the new tubes. The supernatant was mixed with 300 μl of chloroform and 300 μl of ultra performance liquid chromatography (UPLC) grade water and then centrifuged at 20,817 × g for 2 min. After that, 100 μl of the water/methanol phase was dried in a vacuum concentrator (Eppendorf Concentrator Plus) for derivatization [77] for GC/MS analysis. The remaining water/methanol phase was transferred to UPLC vials for LC/MS analysis.

### Sap sampling

Xylem sap was extracted from a 15-cm branch using the pressure chamber technique according to the following methodology [78]: after the balancing pressure was reached, the cut surface was blotted dry. Initially, the pressure was applied slowly at a rate of 0.03 MPa/min until the first droplets of xylem sap reached the surface of the cut. The first sap droplets were discarded to avoid contamination from damaged tissues. The pressure was then slightly increased (0.1 MPa) at a constant rate of 5 kPa/s. In branches obtained from plants subjected to the water deficit treatment, collection was harder. In the event that insufficient sap was collected (< 50 μl), the sap of an additional branch was collected in the same manner. Following sampling, sap was immediately frozen and stored at -80°C until further analysis by LC/MS.

### GC/MS derivatization, data processing

GC/MS analysis samples were processed essentially as described in [77,79]. Residues were redissolved and derivatized for 120 min at 37°C (in 40 μL of 20-mg/mL methoxyamine hydrochloride in pyridine) followed by a 30-min treatment with 70 μL *N*-methyl-*N*-(trimethylsilyl) trifluoroacetamide at 37°C. Eight microliters of a retention time standard mixture (0.029% v/v *n*-dodecane, *n*-pentadecane, *n*-nonadecane, *n*-docosane, *n*-octacosane, *n*-dotracontane, and *n*-hexatriacontane dissolved in pyridine) was added prior to trimethylsilylation. The sample set also included an *Arabidopsis thaliana* quality control reference from a bulked extraction of Columbia-0 plants and a mixture of authentic metabolite standards (0.05 mg/ml).

Sample volumes of 1 μl were then injected into the GC column. The GC/MS system consisted of an AS 3000 autosampler, a TRACE GC ULTRA gas chromatograph, and a DSQII quadrupole mass spectrometer (Thermo-Fisher ltd). The mass spectrometer was tuned according to the manufacturer’s recommendations using tris-(perfluorobutyl)-amine (CF43). GC was performed on a 30-m VF-5 ms column with 0.25 mm i.d., film thickness of 0.25 μm, and + 10 m EZ-Guard (Agilent). A 1-μl sample was injected into an injection port liner (Split liner with Wool, Restek, USA). The use of a programmable temperature vaporizer (PTV) enabled control of the injection temperature gradient from 60°C to 300°C at a rate of 14.5°C/s, the transfer line was 300°C, and the ion source was adjusted to 250°C. Helium set at a constant flow rate of 1 ml/min was the carrier gas. The temperature program comprised 1 min of isothermal heating at 70°C, a 1-°C/min oven temperature ramp to 76°C, then a 6-°C/min oven temperature ramp to 350°C, and finally, 5 min of heating at 350°C.

Mass spectra were recorded at eight scans per second with a mass-to-charge ratio 70 to 700 scanning range. Acquired spectra were then searched for using the National Institute of Standards and Technology (NIST, Gaithersburg, USA) algorithm incorporated in the Xcalibur® data system (version 2.0.7) against RI libraries downloadable from the Max-Planck Institute for Plant Physiology in Golm, Germany (http://gmd.mpimp-golm.mpg.de/) and finally normalized by the internal standard ribitol and the relative water content of the tissue. Amino acids were quantified using calibration curves of standards (Sigma-Aldrich) based on 18 reference points in the range of 12.5-2200 ng. We used the spiking technique according to Kopka et al. [80] to distinguish between metabolites with very similar retention indexes and spectra (e.g., rhamnose and fucose). Since metabolite concentrations spanned more than five orders of magnitude, the splitless injection method used in the study permitted their identification, but it could not be used for the absolute quantification of highly abundant metabolites (e.g., sugars). The measurements of a few metabolites that were outside linear correlation (sugars, inositol) may underestimate the actual change in their levels. In places where the same molecule presented different trimethylsilyl (tms) derivatives with similar patterns of change, only one representative of the group was shown. When there was no agreement in the behavior of the different tms derivatives, we presented both metabolites (e.g., Phe 1tms and 2tms).

### LC/MS analysis

For LC/MS analysis, 4 μl of extracted sample was injected into a Xevo™ QTOF in combination with the Waters Acquity UPLC System and equipped with an ESI interface (Waters MS Technologies, Manchester, UK) operating in negative ion mode. Chromatographic separation was carried out on an Acquity UPLC BEH C_18_ column (100 mm × 2.1 mm, 1.7 μm). The column and autosampler were maintained at 40°C and 10°C, respectively. To ensure accuracy and reproducibility, all analyses were carried out using leucine enkephalin for lock mass calibration at a concentration of 0.4 ng/L, in 50/50 ACN/H_2_O with 0.1% v/v formic acid. The MS conditions were set as follows: Capillary voltage +3.0 keV; sampling cone voltage 27 V; extraction cone voltage 4 V; source temperature: 120°C; desolvation temperature: 300°C; cone gas flow: 50 L/h; desolvation gas flow: 650 L/h; collision energy: 6 eV, and for MS/MS spectra, collision energies were set from 25 to 50 eV; the scan range was set at 50–1500 m/z; and the dynamic range enhancement mode was off. During the running of each sample, the mobile phase comprised 95% water, 5% acetonitrile, 0.1% formic acid (phase A), and 0.1% formic acid in acetonitrile (phase B). The solvent gradient program was conditioned at 100-60% solvent A over the first 8 min, 60-0% solvent A over the next 1 min, and a return to the initial 100% A for 3.5 min, and conditioning at 100% A for 2.5 min, such that a single run comprised 15 min.

### LC/MS data processing

MassLynxTM software (Waters) version 4.1 was used for system control and data acquisition. The raw data acquired were processed using the MarkerLynx application manager (Waters), and the ion intensities for each peak detected were then normalized to 10,000, within each sample, to the sum of the peak intensities in that sample. Metabolites were annotated based on fragmentation patterns searched against in the Chemspider metabolite database (http://www.chemspider.com/), and the consistency of their retention times with those of identified metabolites was compared with the data in the current scientific literature.

When the same metabolite was detected in both the GC/MS and the LC/MS, we referred only to a representative value in the results section, though both values were presented. Conversely, when GC/MS and LC/MS results diverged, the lack of agreement was discussed in the text.

### Statistical analysis

At each sampling day the irrigation treatments were compared for each cultivar independently, to test the significant changes of each variety in response to drought. Results from irrigated treatment were compared between the cultivars to estimate the extent of variety dependent differences in leaf metabolism under well-watered conditions. At the first time point, 4th day prior to irrigation, values of 12 plants per cultivar were averaged. Later time points included 6 plants per each cultivar and condition.

Student’s *t*-test (p-value < 0.05) was performed to determine statistically significant differences between means and to estimate the effects of treatment and cultivar on metabolite abundance. Statistical tests were performed using R 3.0.1. A multiple hypothesis correction using a false discovery rate at a Q value of 0.05 was applied. Differences in metabolite abundance between treatments or between genotypes were considered statistically valid only if (i) the same trend was measured across all sampling days and (ii) significant differences were shown at a minimum of one time point. Differences that were statistically significant in the GC/MS dataset but not in the LC/MS dataset or vice versa (e.g., tartarate) were considered only if they showed similar patterns of change in the two datasets. Cultivar differences and stress response are presented separately in the following sections.

Principle component analysis was run using TMev: Multi Experiment Viewer [81] on logarithmically normalized data (base 10). The Extended Statistics (XS) module of the EZinfo software (Waters LTD) was used to perform multivariate statistical analysis of the LC/MS dataset. Orthogonal Partial Least Squares Discriminate Analysis (OPLS-DA) with Pareto scaling was used to identify variables that are responsible for separation between groups and to select potential molecular markers.

### Correlation-based network analysis

Correlation analyses between all metabolite pairs and between metabolite and physiological trait pairs were performed using the Pearson’s product–moment correlation (Pearson’s ρ) on each of the four matrices of data profiles obtained from the two cultivars (Shiraz and Cabernet Sauvignon) under the two conditions (water deficit and irrigated). To reconstruct a network that would capture the coordinated changes in the metabolic profiles from each of the four data matrices, we first determined threshold values for Pearson’s correlation coefficient that would ensure a q-value of 0.01.

All computations and network visualizations were generated in R. Cytoscape [82] version 2.8.3 was used for network visualization. Network properties and communities were computed by the igraph R package. The following network properties were investigated: average node degree, defined as the average number of edges per node in a network; clustering coefficient, quantifying the local cohesiveness of a network characterized by the extent to which the neighbors of a node are mutually connected; network density, characterizing the proportion of edges in a network in relation to the total number of all possible edges in a network; and diameter, given by the longest path among all shortest paths over all pairs of nodes present in the network [83].

To conduct a comparative analysis, we determined the network intersection between the irrigated and water deficit treatments, between Cabernet Sauvignon and Shiraz, and the network symmetric difference between the two treatments for each of the cultivars. The network differences were used to establish the extent to which a particular metabolite contributed to the treatment-specific relationships included in the reconstructed networks. The water-deficit-specific contribution of a metabolite was quantified by the ratio of the degree of the corresponding node in the water-deficit/irrigated network difference and the degree of the node in the water deficit network. Analogously, the irrigated-specific contribution of a metabolite was quantified by the ratio of the degree of the corresponding node in the irrigated/water deficit network difference and the degree of the node in the irrigated network.

To determine the statistical significance level of network parameters discerns, we performed empirical p-value estimations via permutation tests. Each metabolite for each variety under both irrigation regimes, i.e. Cabernet Sauvignon irrigated, Cabernet Sauvignon water deficit, Shiraz irrigated, Shiraz water deficit, was permuted individually. Subsequently, the shuffled datasets were used for pairwise correlation analysis as described before. To identify significant correlations, the same permissive r- and q-values thresholds as outlined above were applied (*r* > =0.9 and q < =0.01). The resulting adjacency matrices were used to construct correlation-based networks followed by network parameter estimations. This test was repeated with 1,000 iterations. At each iteration, the differences of network parameters for each variety between the different irrigation regimes were computed. A counter (*c*) was set to monitor the number of times the differences of the permutation test equaled or exceeded the initially observed value. To estimate the p-value of the initially observed discerns between the two irrigation treatments, the following equation was used:

$$p=\frac{c+1}{1000+1}$$

To demonstrate the differences in the connections between metabolites of similar biochemical backgrounds, we ordered them into communities of compound classes. To characterize the coordination between metabolic processes, we conducted enrichment analyses with respect to the classes of compounds present in each of the identified communities.

### Availability of supporting data

The data sets supporting the results of this article are included within the article and its additional files.

## Authors’ contribution

UH conceived and conducted the experiment, analyzed GCMS data and wrote the body of the paper with AF; AD helped running the experiment, processed the samples for metabolite profiling and analyzed LCMS data. TG investigated the microscopy based analysis of suberin deposition. DT and ZN carried the CNA and assisted with the statistical analyses and writing of the MS. AF conceived and coordinated the project together with SR. All authors reviewed, edited and approved the final version of the manuscript.

## Supplementary Material

Additional file 1: Table S1 Volumetric soil water content of Cabernet Sauvignon (Cs) and Shiraz (Sh) irrigated (IR) vs. water deficit (D) treatment. Net assimilation (A_N_), stomatal conductance (g_s_), midday leaf water potential (Ψ_l_) and Osmolality (π) on days 4, 18, 26, and 34. **Table S2.** Statistical data of the PCA (principle component analysis) of GC/MS and LC/MS. Loading (A) and % of variance explained (B) of the different components are presented. **Table S4.** Six network properties (number of nodes, number of edges, average nodal degree, network density, clustering coefficient, and network diameter calculated for the water deficit (D) and irrigated (IR) treatment networks of Cabernet Sauvignon (Cs) and Shiraz (Sh) and for their respective network unions, intersects, differences, and symmetric differences. **Table S5.** Ratios of the nodal degree of each metabolite between the treatment network intersects of the cultivars and the nodal degree of the corresponding metabolite in the respective complete treatment-specific network, i.e., for each cultivar and treatment individually. **Table S6.** Metabolites (nodes) and corresponding nodal degrees in descending order and according to cultivar and treatment Average r coefficient of nodes vs. the physiological traits is also shown. Click here for file

Additional file 2: Figure S1 Correlation of Ψ_l_ to the total osmolite concentration (A) and to the relative abundance of Proline (B), Valine (C) and Leucine (D) in Shiraz and Cabernet Sauvignon. **Figure S2.** PCA plot (x-1st component, y-3rd component) of Cabernet Sauvignon and Shiraz grape leaf extracts of GC/MS based metabolites **Figure S3.** S-plot of the OPLS-DA model from Shiraz irrigated vs. water deficit treatments of leaf sample metabolite markers analyzed in negative ESI mode on day 34 of the experiment. **Figure S4.** S-plot of the OPLS-DA model from Cabernet Sauvignon irrigated vs. water deficit treatments of leaf sample metabolite markers analyzed in negative ESI mode on day 34 of the experiment. **Figure S5.** Correlation of stomatal conductance (g_s_) to Abscisic acid (ABA) – as measured in the sap – in Shiraz and Cabernet Sauvignon. **Figure S6.** Symmetric difference network based on correlation between irrigated treatment and metabolites. **Figure S7.** Symmetric difference network based on correlation between water deficit treatment and metabolites. Click here for file

Additional file 3: Table S3a Relative metabolite content (GC/MS) in leaves of Shiraz (Sh) and Cabernet Sauvignon (Cs) grown under irrigated (IR) and water deficit (D) conditions. **Table S3b.** Relative metabolite content (LC/MS) in leaves of Shiraz (Sh) and Cabernet Sauvignon (Cs) grown under irrigated (IR) and water deficit (D) conditions. **Table S3c.** Relative metabolite content (LC/MS) in sap of Shiraz (Sh) and Cabernet Sauvignon (Cs) grown under irrigated (IR) and water deficit (D) conditions. Click here for file
