# Supplementary material for: Metabolite profiling and network analysis reveal coordinated changes in grapevine water stress response
Source: BMC Plant Biol. 2013 Nov 20;13:184. doi: 10.1186/1471-2229-13-184 (PMC4225576; doi:10.1186/1471-2229-13-184)

## Slide 1
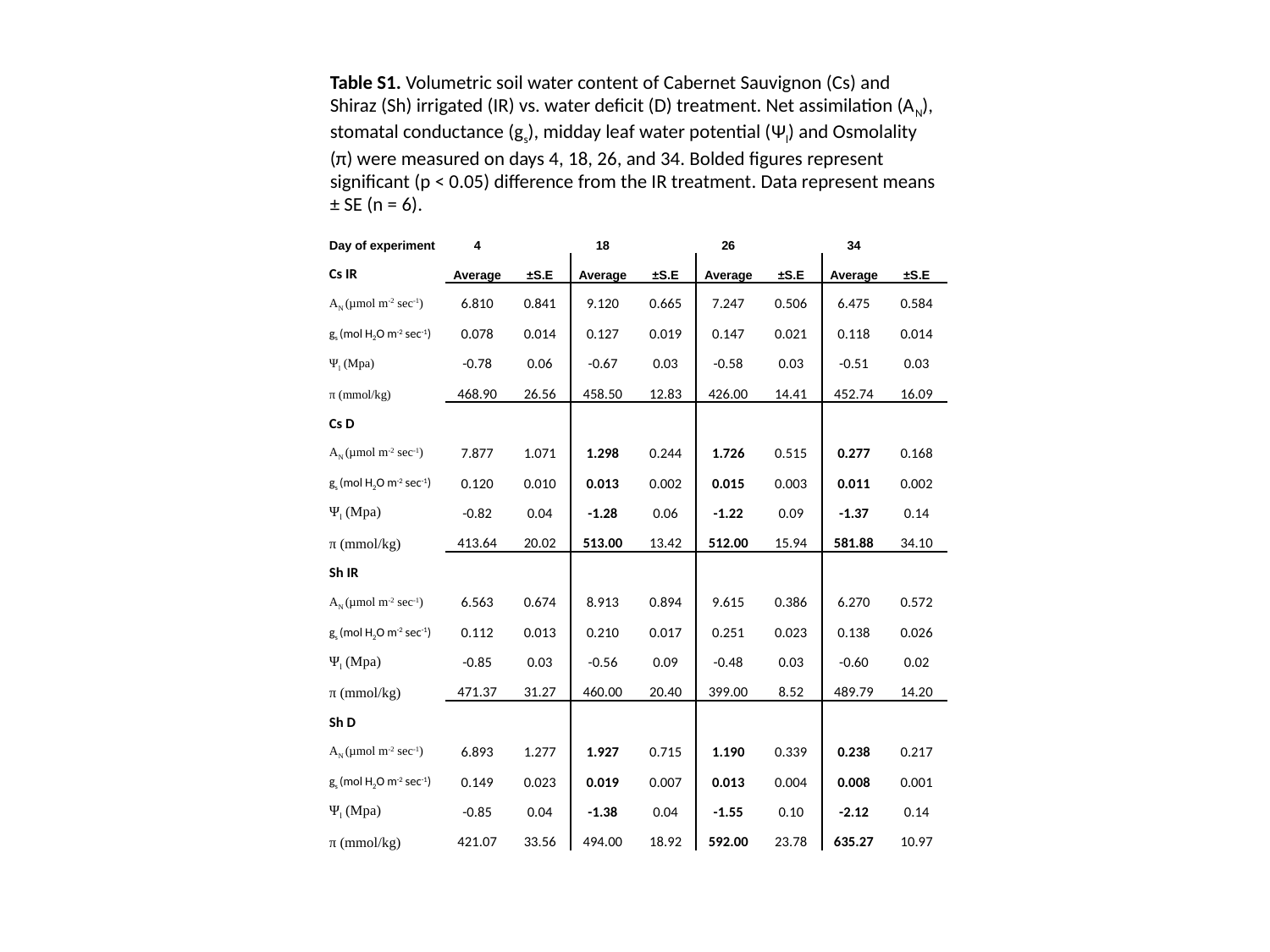

Table S1. Volumetric soil water content of Cabernet Sauvignon (Cs) and Shiraz (Sh) irrigated (IR) vs. water deficit (D) treatment. Net assimilation (AN), stomatal conductance (gs), midday leaf water potential (Ψl) and Osmolality (π) were measured on days 4, 18, 26, and 34. Bolded figures represent significant (p < 0.05) difference from the IR treatment. Data represent means ± SE (n = 6).
| Day of experiment | 4 | | 18 | | 26 | | 34 | |
| --- | --- | --- | --- | --- | --- | --- | --- | --- |
| Cs IR | Average | ±S.E | Average | ±S.E | Average | ±S.E | Average | ±S.E |
| AN (µmol m-2 sec-1) | 6.810 | 0.841 | 9.120 | 0.665 | 7.247 | 0.506 | 6.475 | 0.584 |
| gs (mol H2O m-2 sec-1) | 0.078 | 0.014 | 0.127 | 0.019 | 0.147 | 0.021 | 0.118 | 0.014 |
| Ψl (Mpa) | -0.78 | 0.06 | -0.67 | 0.03 | -0.58 | 0.03 | -0.51 | 0.03 |
| π (mmol/kg) | 468.90 | 26.56 | 458.50 | 12.83 | 426.00 | 14.41 | 452.74 | 16.09 |
| Cs D | | | | | | | | |
| AN (µmol m-2 sec-1) | 7.877 | 1.071 | 1.298 | 0.244 | 1.726 | 0.515 | 0.277 | 0.168 |
| gs (mol H2O m-2 sec-1) | 0.120 | 0.010 | 0.013 | 0.002 | 0.015 | 0.003 | 0.011 | 0.002 |
| Ψl (Mpa) | -0.82 | 0.04 | -1.28 | 0.06 | -1.22 | 0.09 | -1.37 | 0.14 |
| π (mmol/kg) | 413.64 | 20.02 | 513.00 | 13.42 | 512.00 | 15.94 | 581.88 | 34.10 |
| Sh IR | | | | | | | | |
| AN (µmol m-2 sec-1) | 6.563 | 0.674 | 8.913 | 0.894 | 9.615 | 0.386 | 6.270 | 0.572 |
| gs (mol H2O m-2 sec-1) | 0.112 | 0.013 | 0.210 | 0.017 | 0.251 | 0.023 | 0.138 | 0.026 |
| Ψl (Mpa) | -0.85 | 0.03 | -0.56 | 0.09 | -0.48 | 0.03 | -0.60 | 0.02 |
| π (mmol/kg) | 471.37 | 31.27 | 460.00 | 20.40 | 399.00 | 8.52 | 489.79 | 14.20 |
| Sh D | | | | | | | | |
| AN (µmol m-2 sec-1) | 6.893 | 1.277 | 1.927 | 0.715 | 1.190 | 0.339 | 0.238 | 0.217 |
| gs (mol H2O m-2 sec-1) | 0.149 | 0.023 | 0.019 | 0.007 | 0.013 | 0.004 | 0.008 | 0.001 |
| Ψl (Mpa) | -0.85 | 0.04 | -1.38 | 0.04 | -1.55 | 0.10 | -2.12 | 0.14 |
| π (mmol/kg) | 421.07 | 33.56 | 494.00 | 18.92 | 592.00 | 23.78 | 635.27 | 10.97 |

## Slide 2
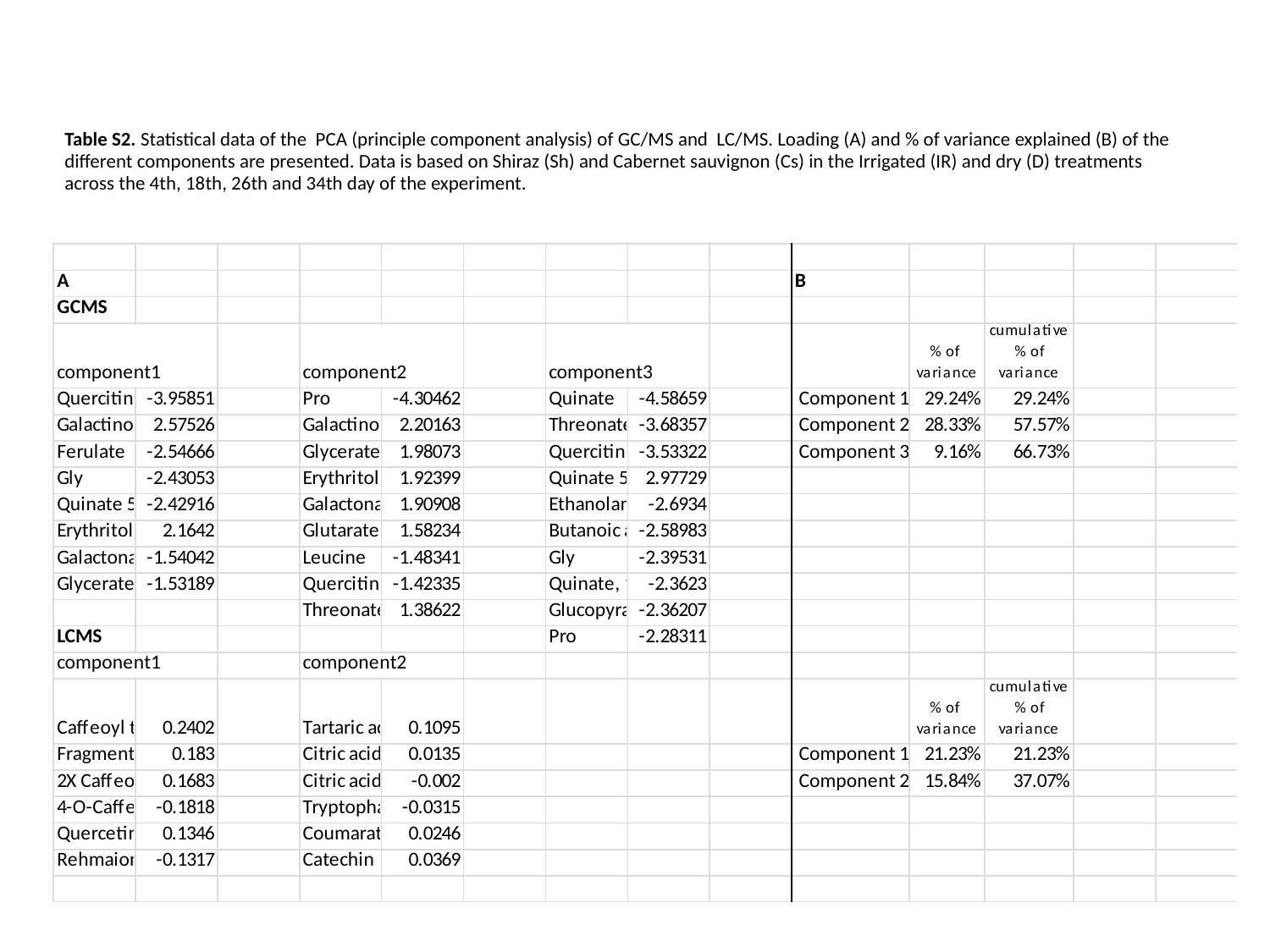

| Table S2. Statistical data of the PCA (principle component analysis) of GC/MS and LC/MS. Loading (A) and % of variance explained (B) of the different components are presented. Data is based on Shiraz (Sh) and Cabernet sauvignon (Cs) in the Irrigated (IR) and dry (D) treatments across the 4th, 18th, 26th and 34th day of the experiment. |
| --- |

## Slide 3
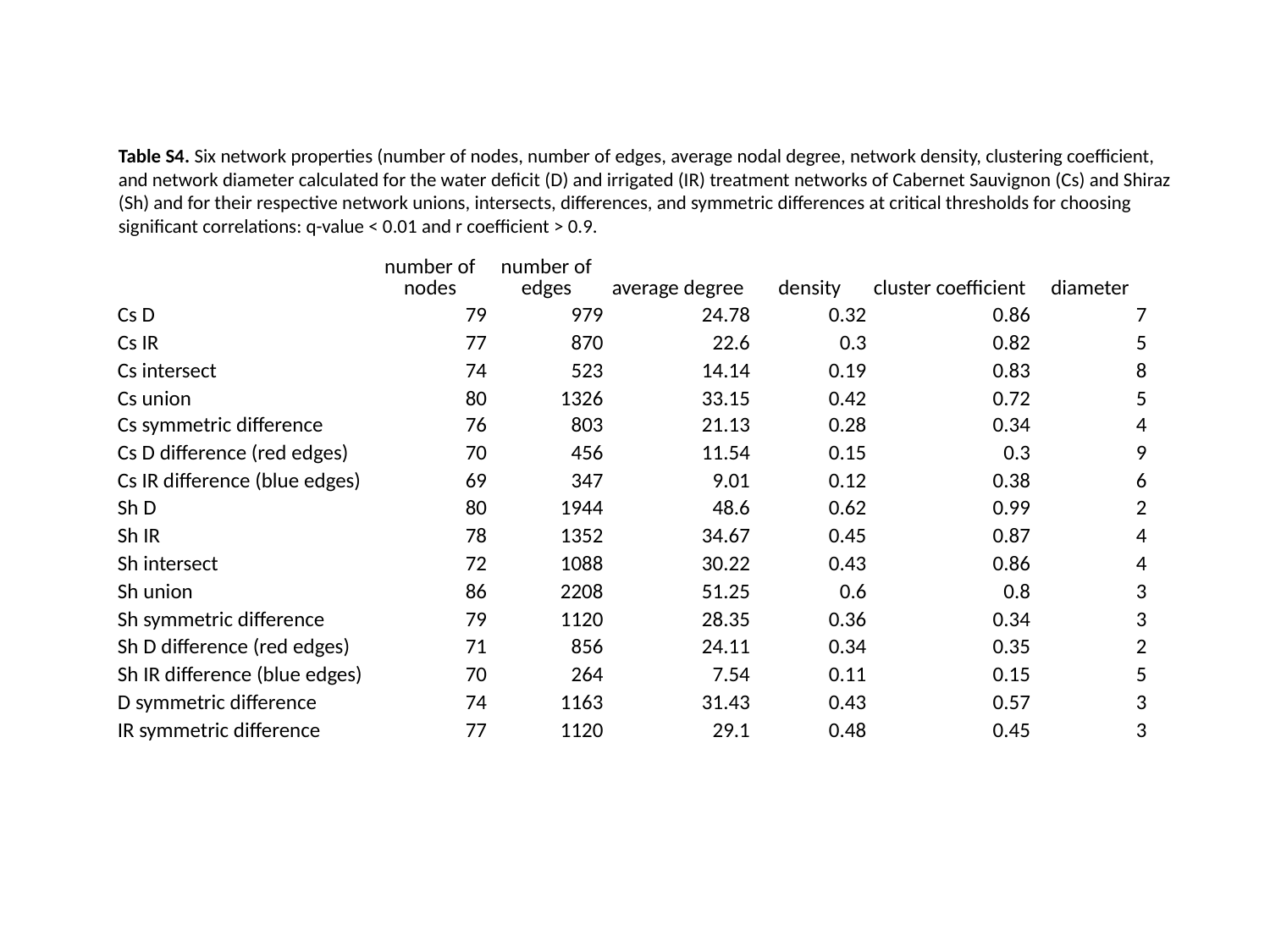

Table S4. Six network properties (number of nodes, number of edges, average nodal degree, network density, clustering coefficient, and network diameter calculated for the water deficit (D) and irrigated (IR) treatment networks of Cabernet Sauvignon (Cs) and Shiraz (Sh) and for their respective network unions, intersects, differences, and symmetric differences at critical thresholds for choosing significant correlations: q-value < 0.01 and r coefficient > 0.9.
| | number of nodes | number of edges | average degree | density | cluster coefficient | diameter |
| --- | --- | --- | --- | --- | --- | --- |
| Cs D | 79 | 979 | 24.78 | 0.32 | 0.86 | 7 |
| Cs IR | 77 | 870 | 22.6 | 0.3 | 0.82 | 5 |
| Cs intersect | 74 | 523 | 14.14 | 0.19 | 0.83 | 8 |
| Cs union | 80 | 1326 | 33.15 | 0.42 | 0.72 | 5 |
| Cs symmetric difference | 76 | 803 | 21.13 | 0.28 | 0.34 | 4 |
| Cs D difference (red edges) | 70 | 456 | 11.54 | 0.15 | 0.3 | 9 |
| Cs IR difference (blue edges) | 69 | 347 | 9.01 | 0.12 | 0.38 | 6 |
| Sh D | 80 | 1944 | 48.6 | 0.62 | 0.99 | 2 |
| Sh IR | 78 | 1352 | 34.67 | 0.45 | 0.87 | 4 |
| Sh intersect | 72 | 1088 | 30.22 | 0.43 | 0.86 | 4 |
| Sh union | 86 | 2208 | 51.25 | 0.6 | 0.8 | 3 |
| Sh symmetric difference | 79 | 1120 | 28.35 | 0.36 | 0.34 | 3 |
| Sh D difference (red edges) | 71 | 856 | 24.11 | 0.34 | 0.35 | 2 |
| Sh IR difference (blue edges) | 70 | 264 | 7.54 | 0.11 | 0.15 | 5 |
| D symmetric difference | 74 | 1163 | 31.43 | 0.43 | 0.57 | 3 |
| IR symmetric difference | 77 | 1120 | 29.1 | 0.48 | 0.45 | 3 |

## Slide 4
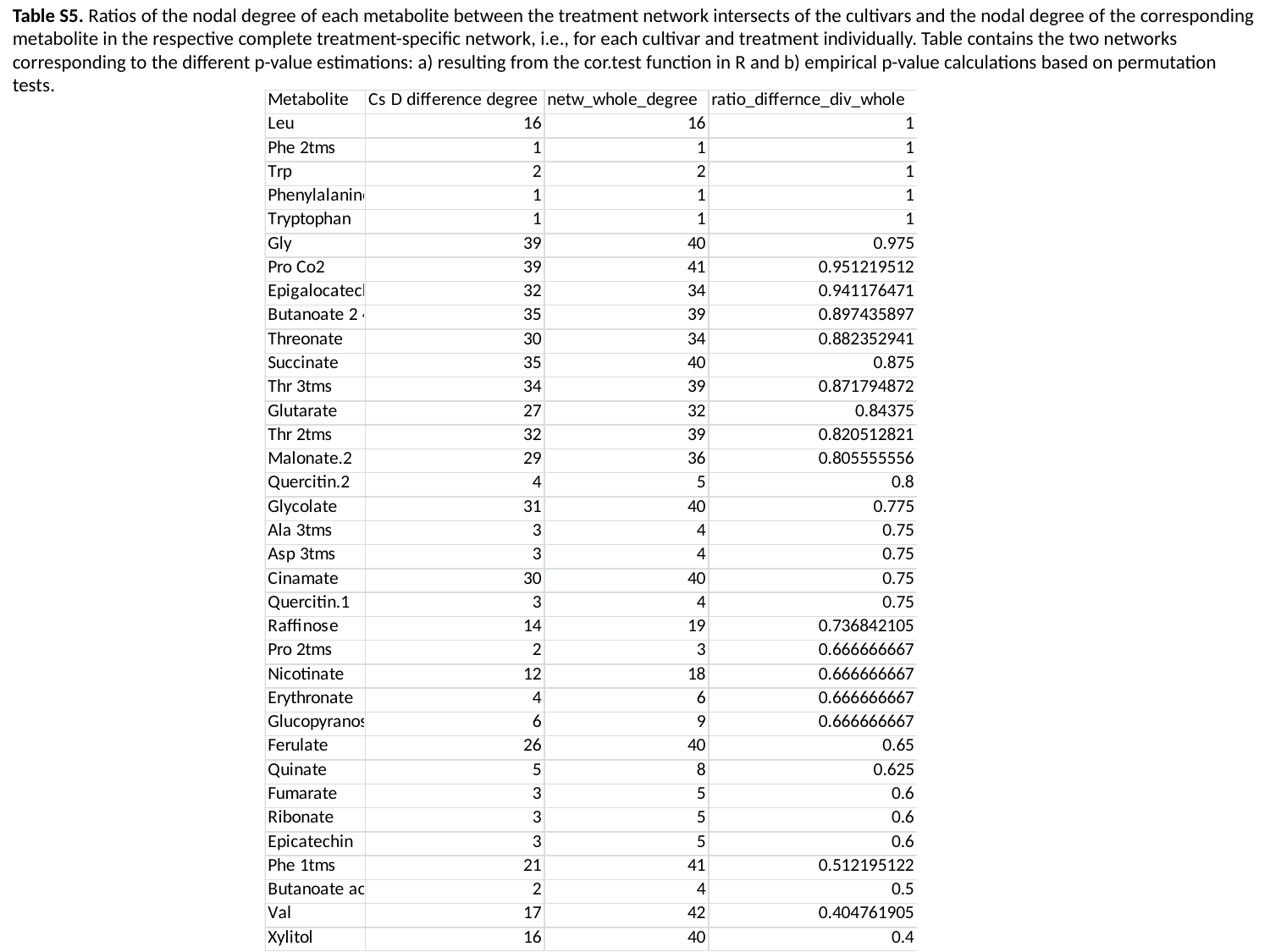

Table S5. Ratios of the nodal degree of each metabolite between the treatment network intersects of the cultivars and the nodal degree of the corresponding metabolite in the respective complete treatment-specific network, i.e., for each cultivar and treatment individually. Table contains the two networks corresponding to the different p-value estimations: a) resulting from the cor.test function in R and b) empirical p-value calculations based on permutation tests.

## Slide 5
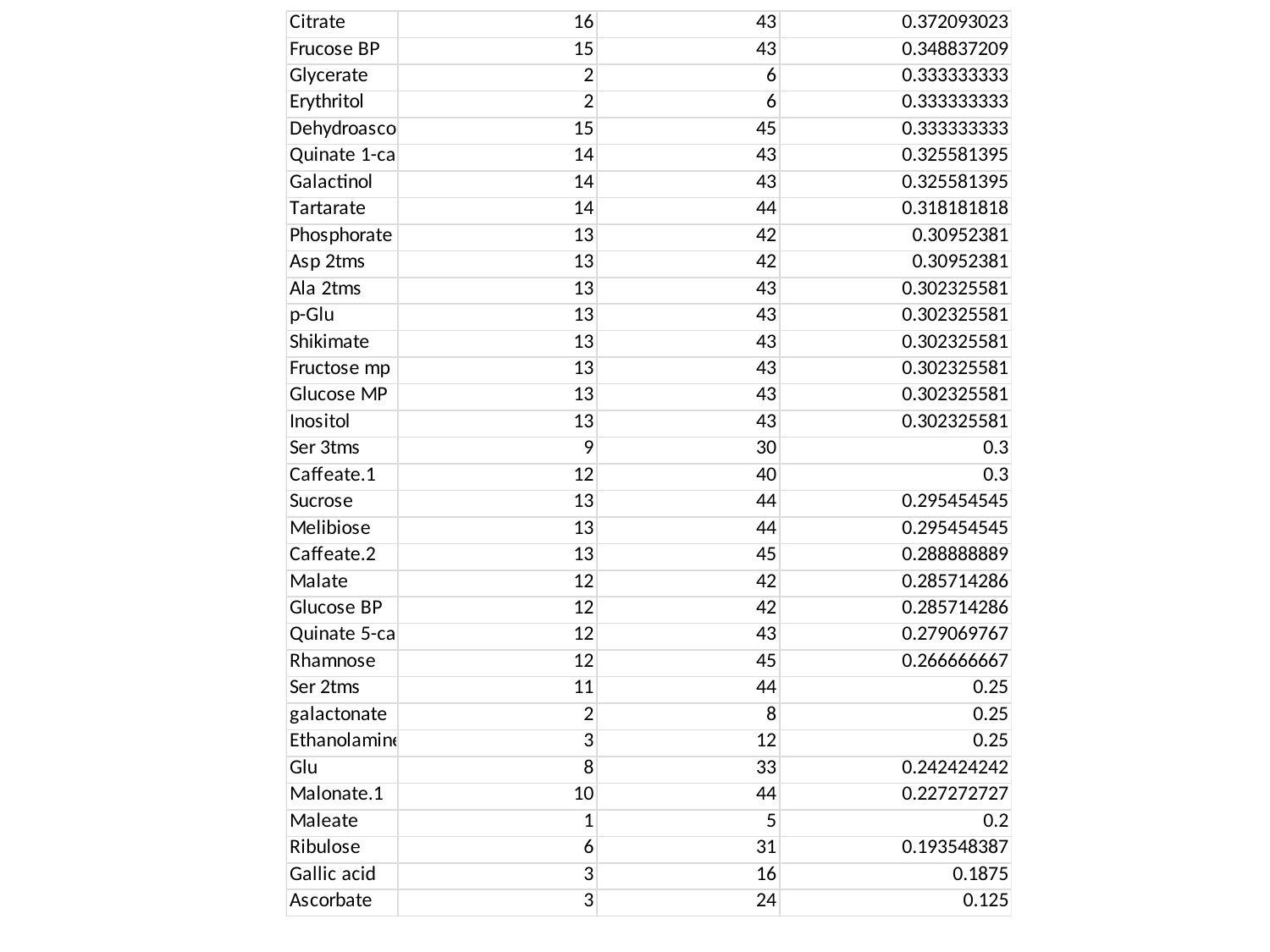

#

## Slide 6
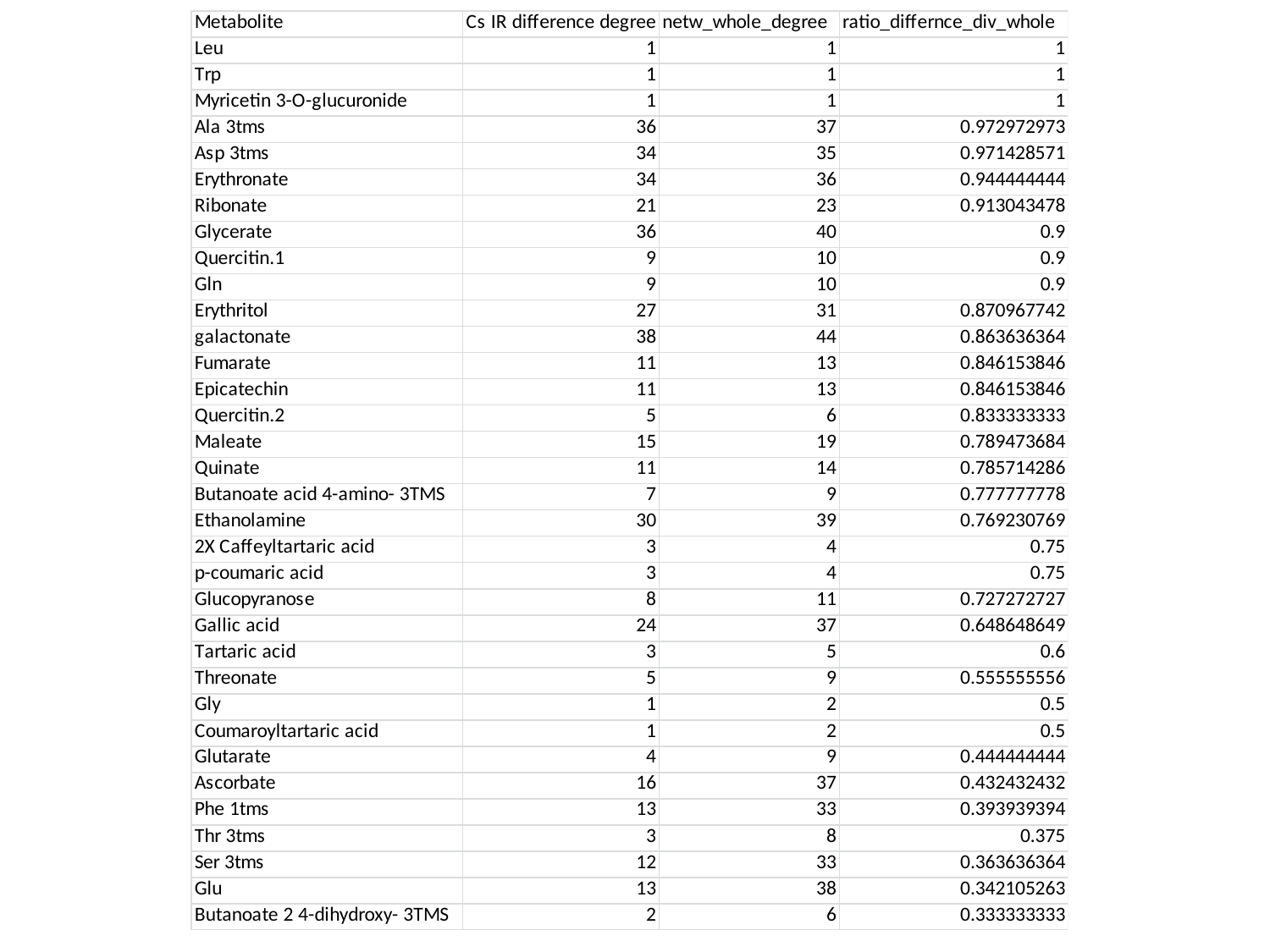

## Slide 7
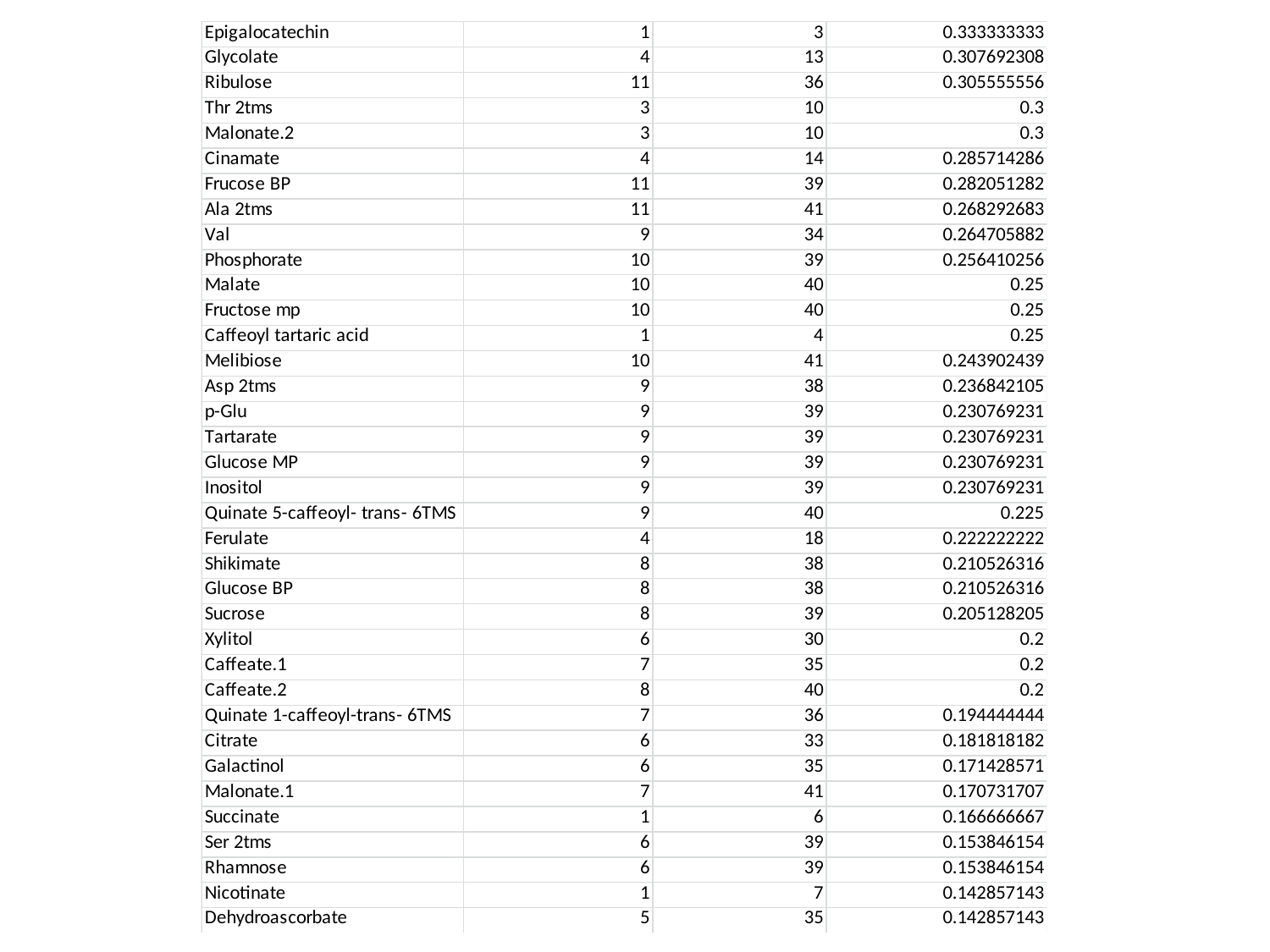

## Slide 8
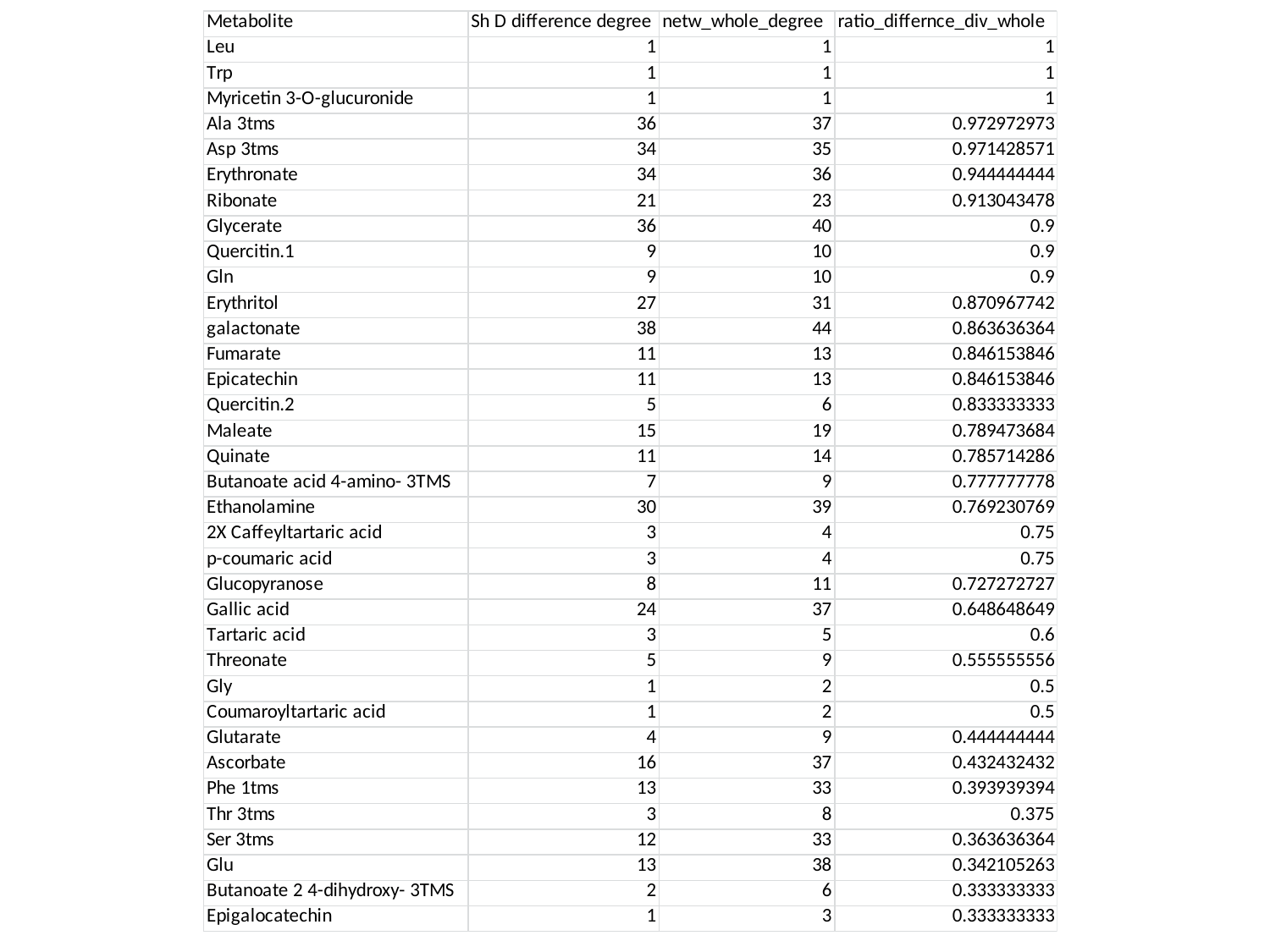

## Slide 9
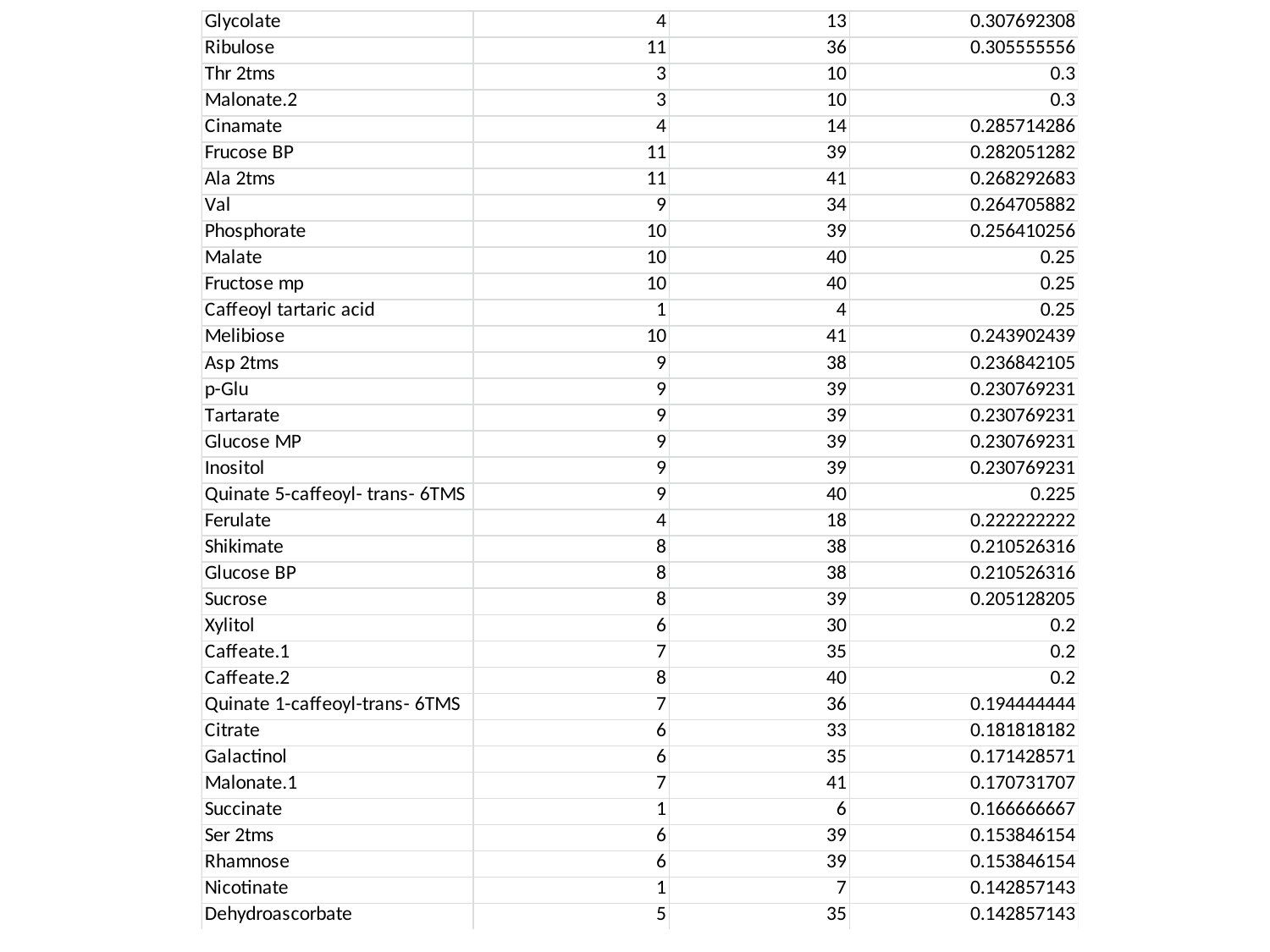

## Slide 10
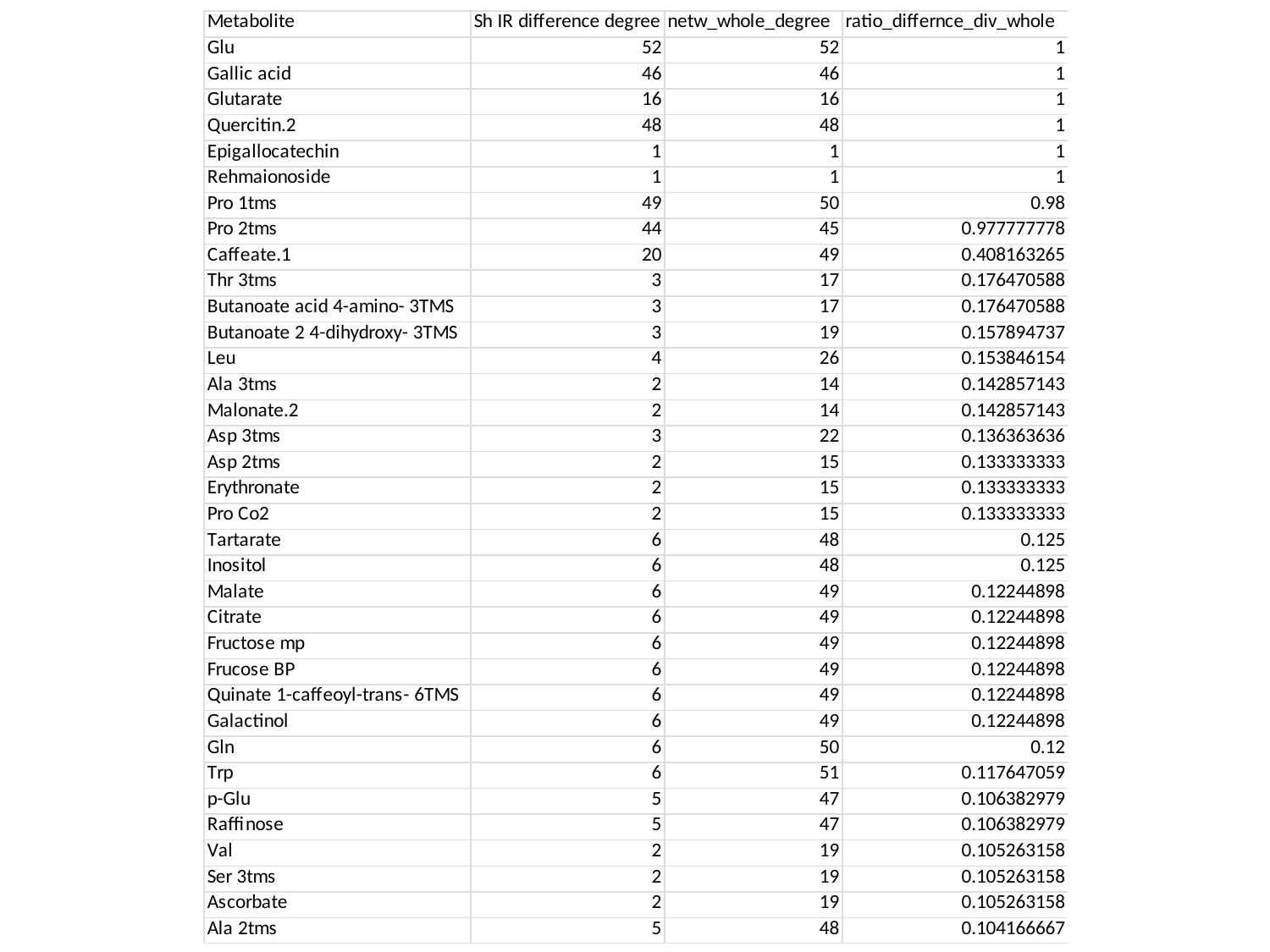

## Slide 11
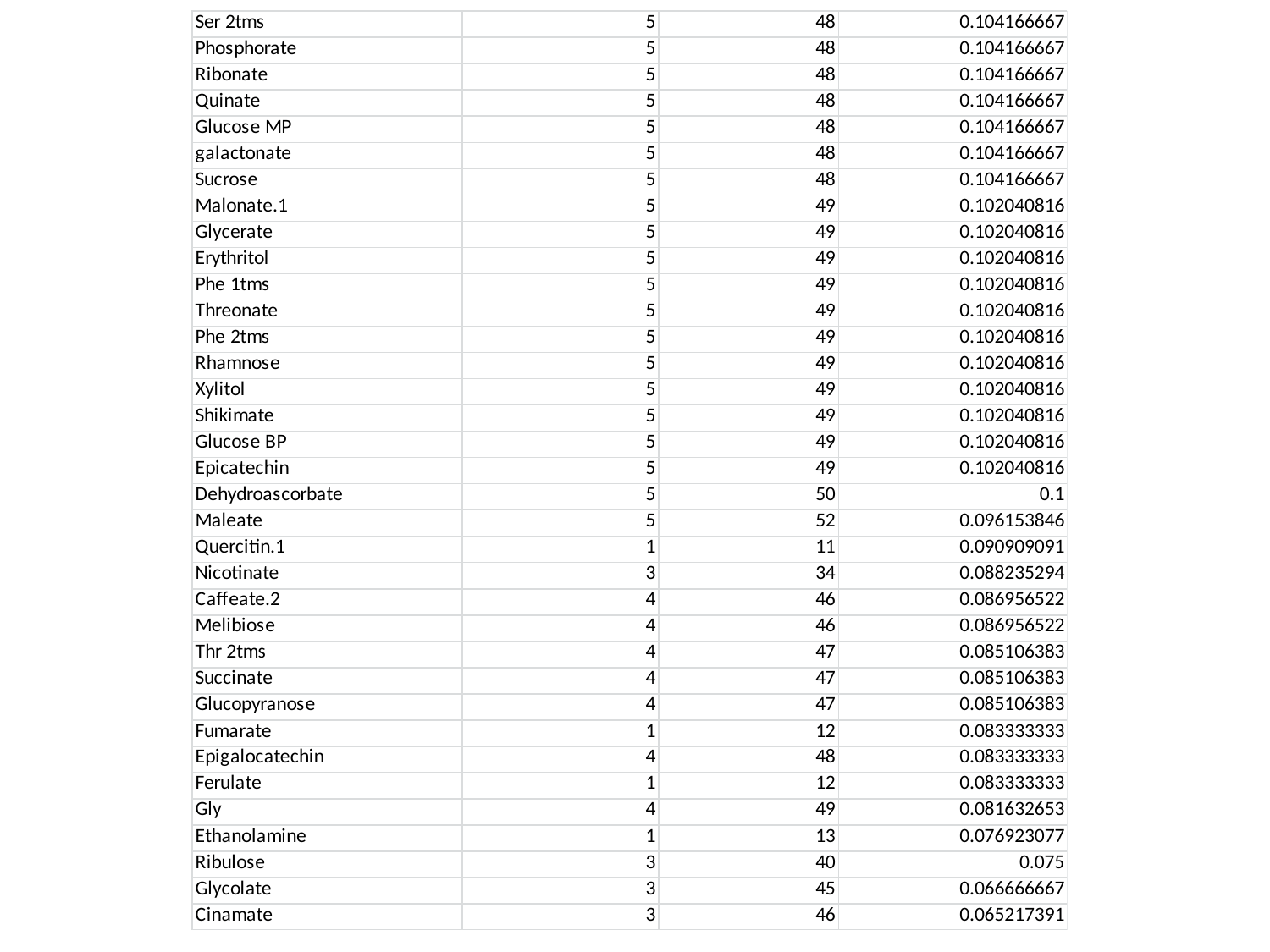

## Slide 12
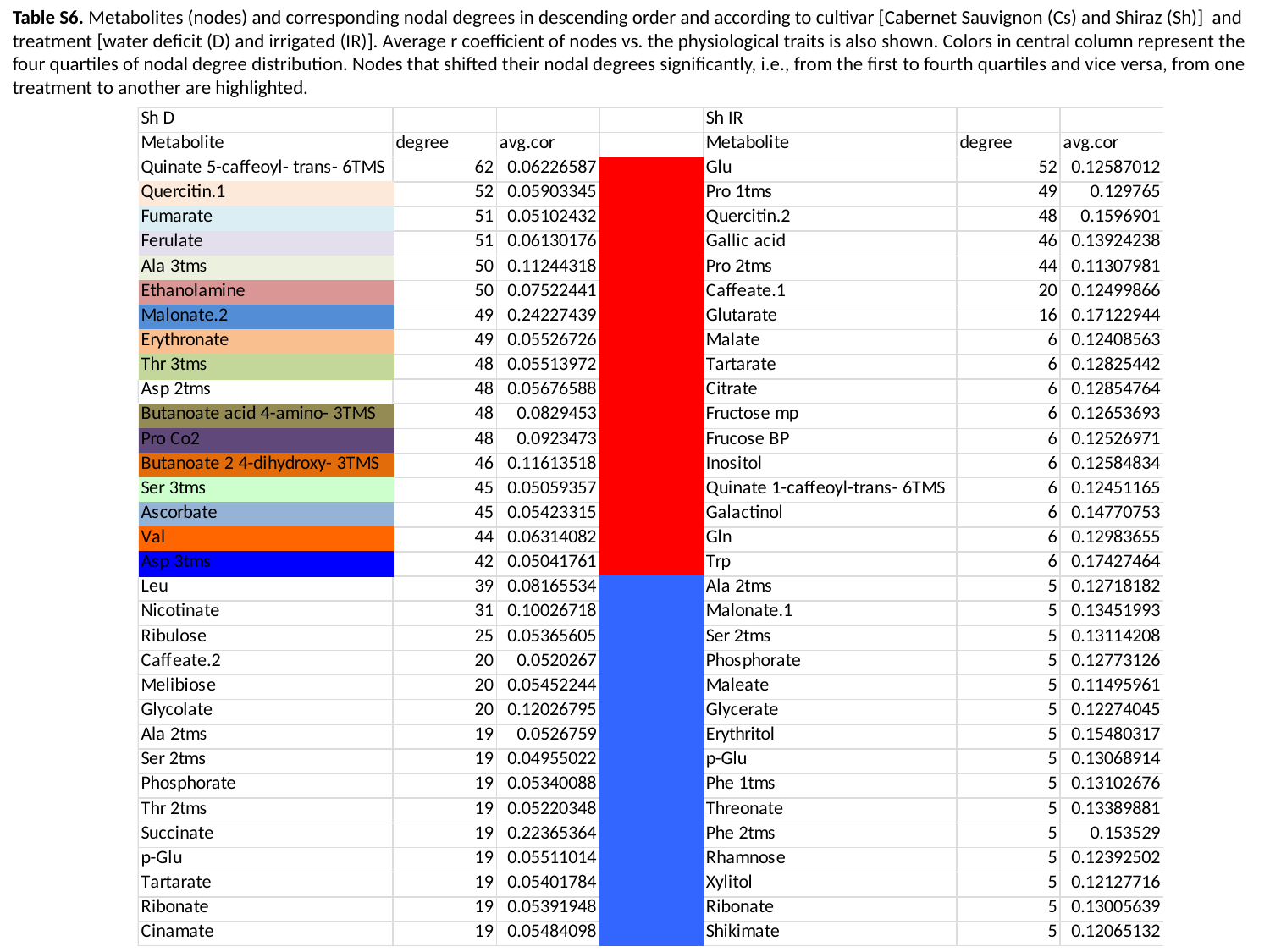

Table S6. Metabolites (nodes) and corresponding nodal degrees in descending order and according to cultivar [Cabernet Sauvignon (Cs) and Shiraz (Sh)] and treatment [water deficit (D) and irrigated (IR)]. Average r coefficient of nodes vs. the physiological traits is also shown. Colors in central column represent the four quartiles of nodal degree distribution. Nodes that shifted their nodal degrees significantly, i.e., from the first to fourth quartiles and vice versa, from one treatment to another are highlighted.

## Slide 13
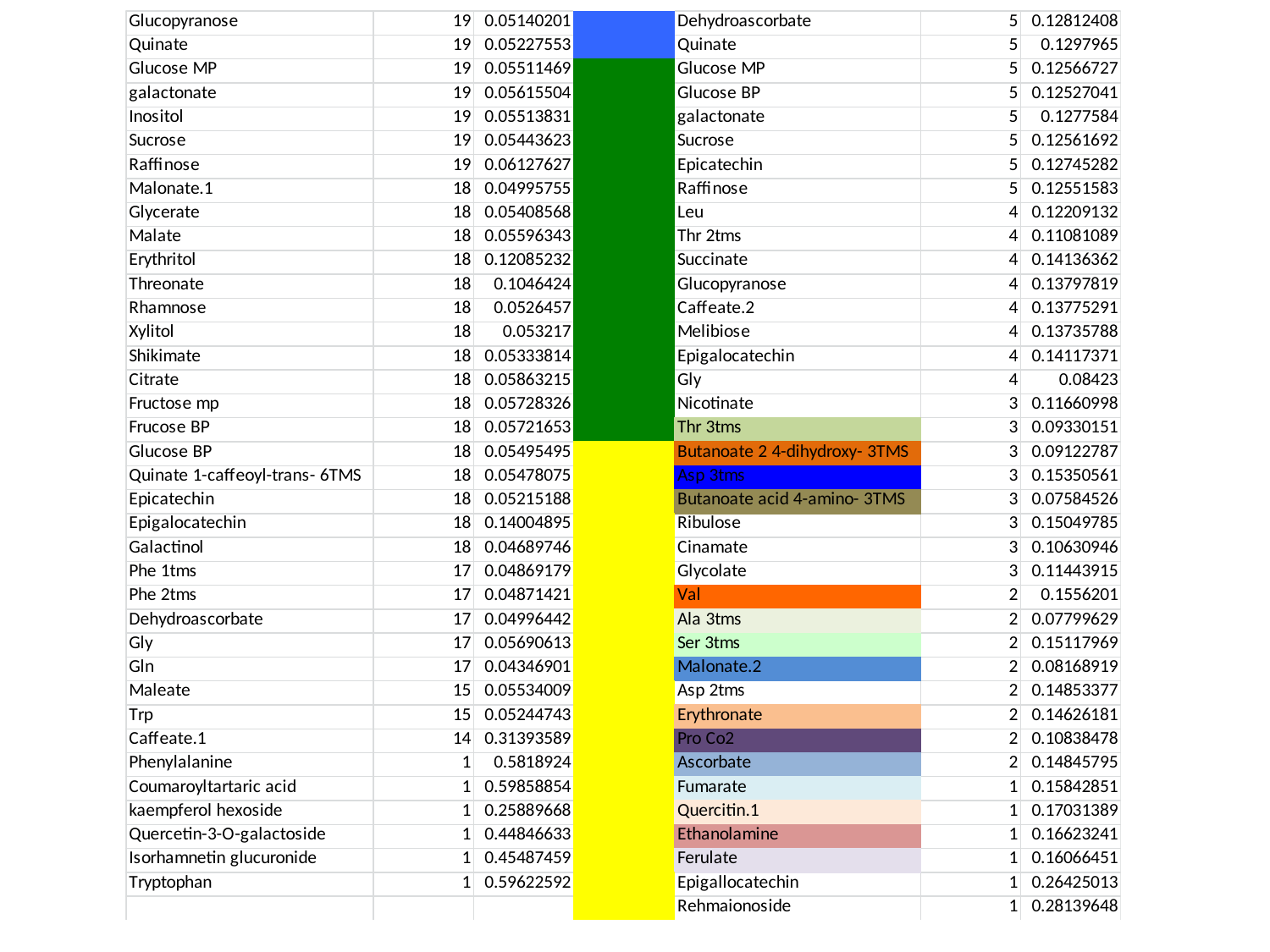

## Slide 14
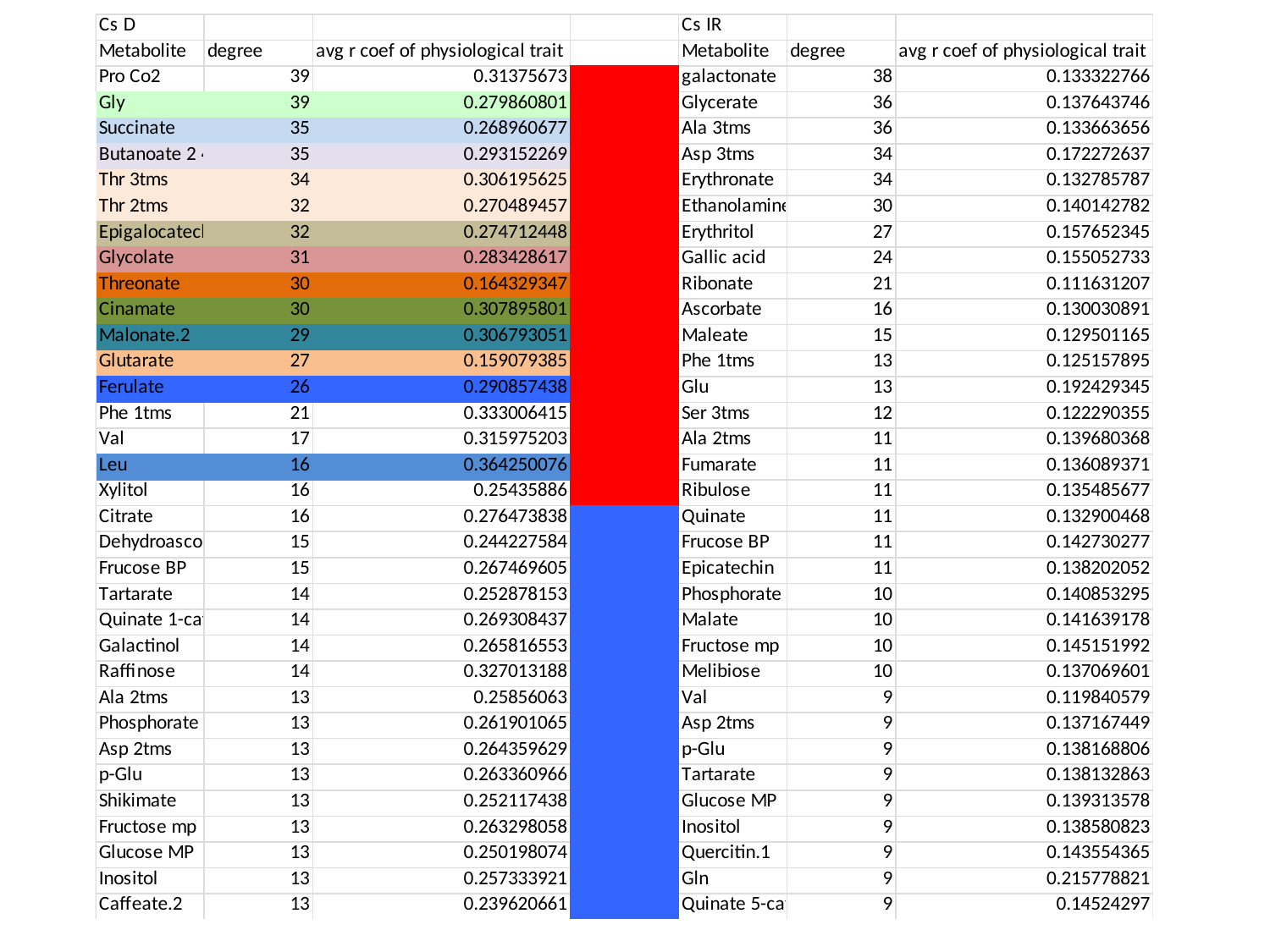

## Slide 15
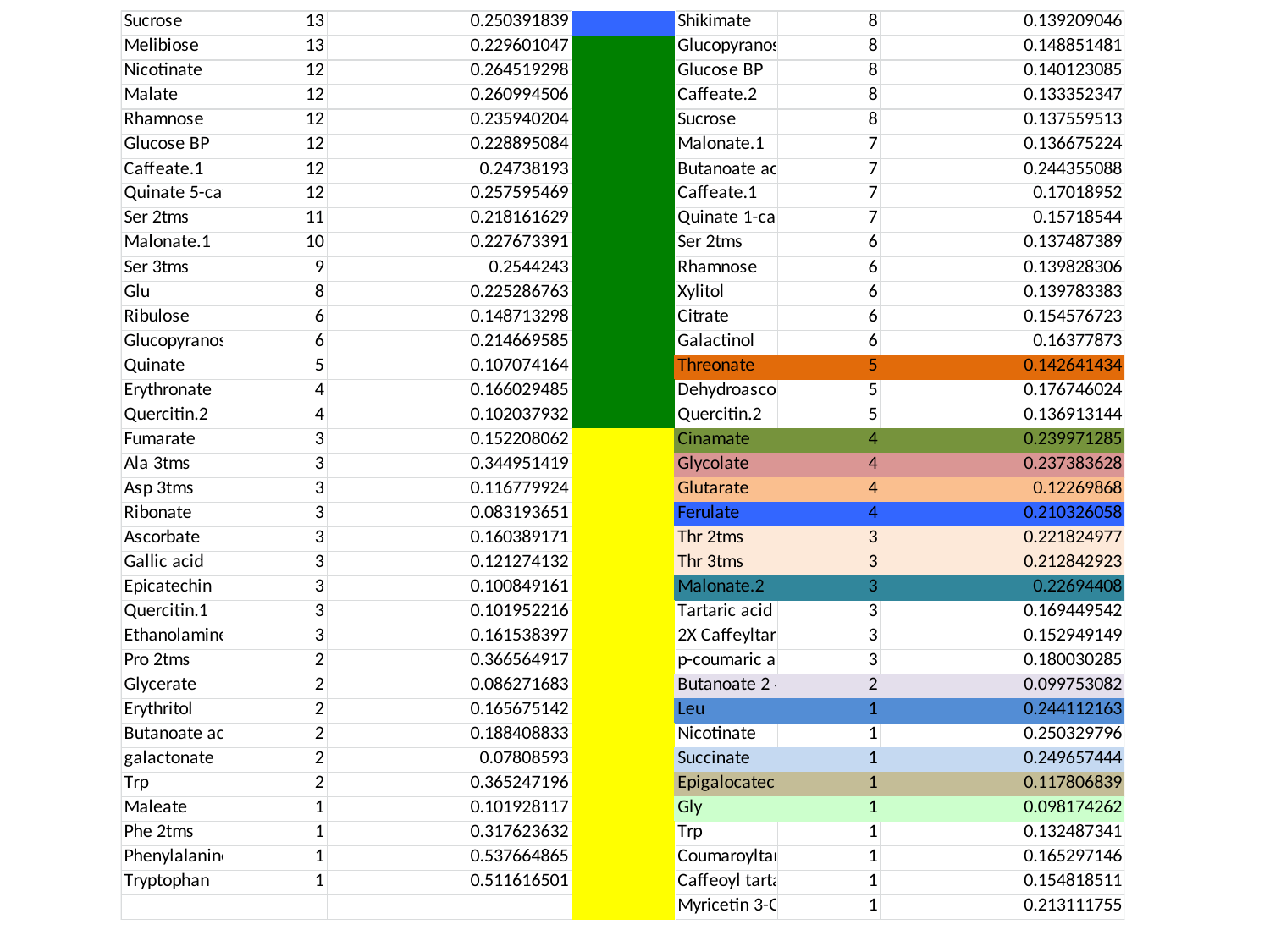

Supplement: Additional file 1: Table S1 — Volumetric soil water content of Cabernet Sauvignon (Cs) and Shiraz (Sh) irrigated (IR) vs. water deficit (D) treatment. Net assimilation (AN), stomatal conductance (gs), midday leaf water potential (Ψl) and Osmolality (π) on days 4, 18, 26, and 34. Table S2. Statistical data of the PCA (principle component analysis) of GC/MS and LC/MS. Loading (A) and % of variance explained (B) of the different components are presented. Table S4. Six network properties (number of nodes, number of edges, average nodal degree, network density, clustering coefficient, and network diameter calculated for the water deficit (D) and irrigated (IR) treatment networks of Cabernet Sauvignon (Cs) and Shiraz (Sh) and for their respective network unions, intersects, differences, and symmetric differences. Table S5. Ratios of the nodal degree of each metabolite between the treatment network intersects of the cultivars and the nodal degree of the corresponding metabolite in the respective complete treatment-specific network, i.e., for each cultivar and treatment individually. Table S6. Metabolites (nodes) and corresponding nodal degrees in descending order and according to cultivar and treatment Average r coefficient of nodes vs. the physiological traits is also shown. [file 1471-2229-13-184-S1.pptx]
