# Supplementary material for: Metabolite profiling and network analysis reveal coordinated changes in grapevine water stress response
Source: BMC Plant Biol. 2013 Nov 20;13:184. doi: 10.1186/1471-2229-13-184 (PMC4225576; doi:10.1186/1471-2229-13-184)
Supplement: Additional file 2: Figure S1 — Correlation of Ψl to the total osmolite concentration (A) and to the relative abundance of Proline (B), Valine (C) and Leucine (D) in Shiraz and Cabernet Sauvignon. Figure S2. PCA plot (x-1st component, y-3rd component) of Cabernet Sauvignon and Shiraz grape leaf extracts of GC/MS based metabolites Figure S3. S-plot of the OPLS-DA model from Shiraz irrigated vs. water deficit treatments of leaf sample metabolite markers analyzed in negative ESI mode on day 34 of the experiment. Figure S4. S-plot of the OPLS-DA model from Cabernet Sauvignon irrigated vs. water deficit treatments of leaf sample metabolite markers analyzed in negative ESI mode on day 34 of the experiment. Figure S5. Correlation of stomatal conductance (gs) to Abscisic acid (ABA) – as measured in the sap – in Shiraz and Cabernet Sauvignon. Figure S6. Symmetric difference network based on correlation between irrigated treatment and metabolites. Figure S7. Symmetric difference network based on correlation between water deficit treatment and metabolites. [file 1471-2229-13-184-S2.pptx]

## Slide 1
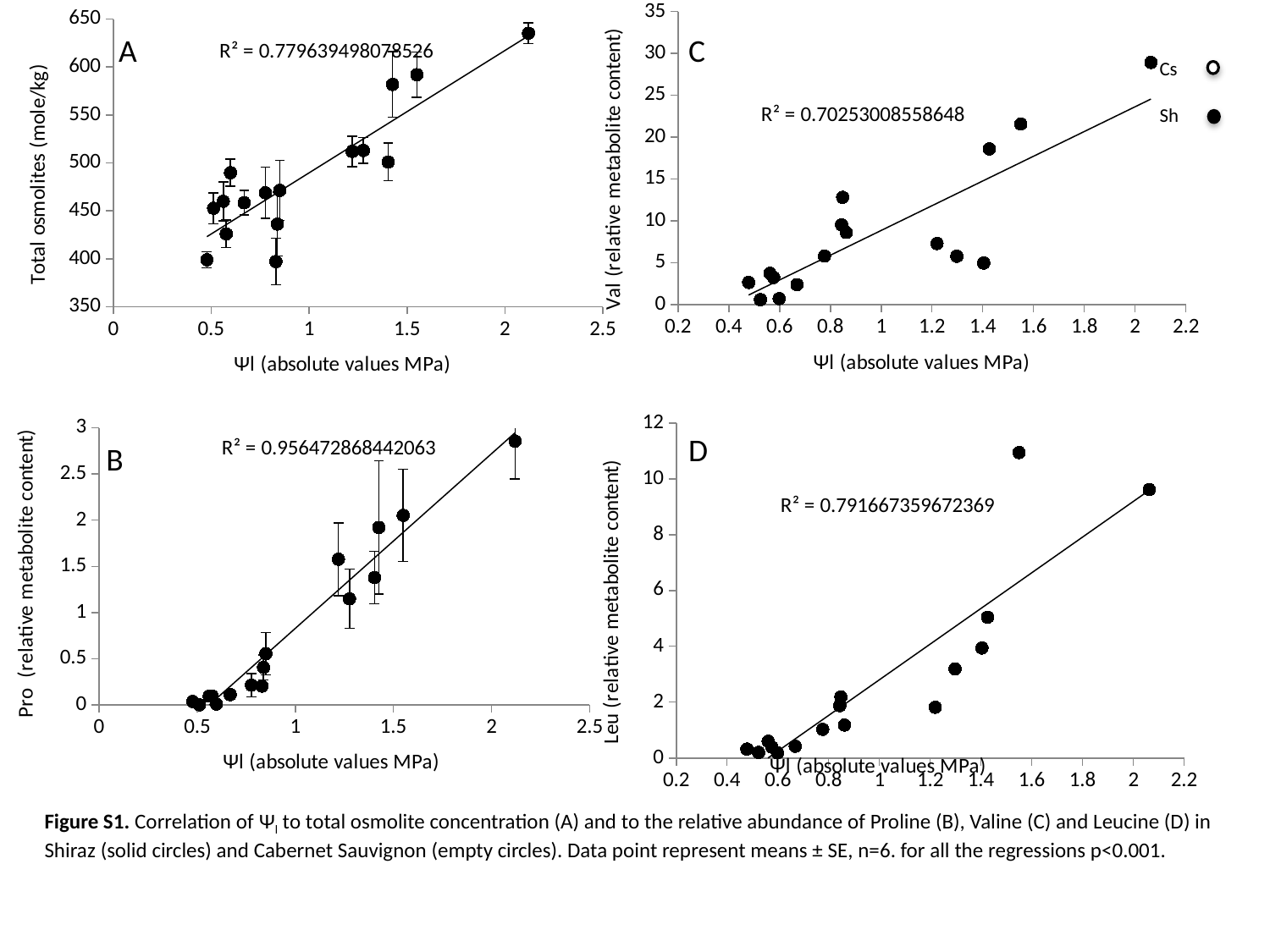

### Chart
| Category | |
|---|---|
### Chart
| Category | |
|---|---|A
C
Cs
Sh
### Chart
| Category | 1264leucine |
|---|---|
### Chart
| Category | |
|---|---|D
B
Figure S1. Correlation of Ψl to total osmolite concentration (A) and to the relative abundance of Proline (B), Valine (C) and Leucine (D) in Shiraz (solid circles) and Cabernet Sauvignon (empty circles). Data point represent means ± SE, n=6. for all the regressions p<0.001.

## Slide 2
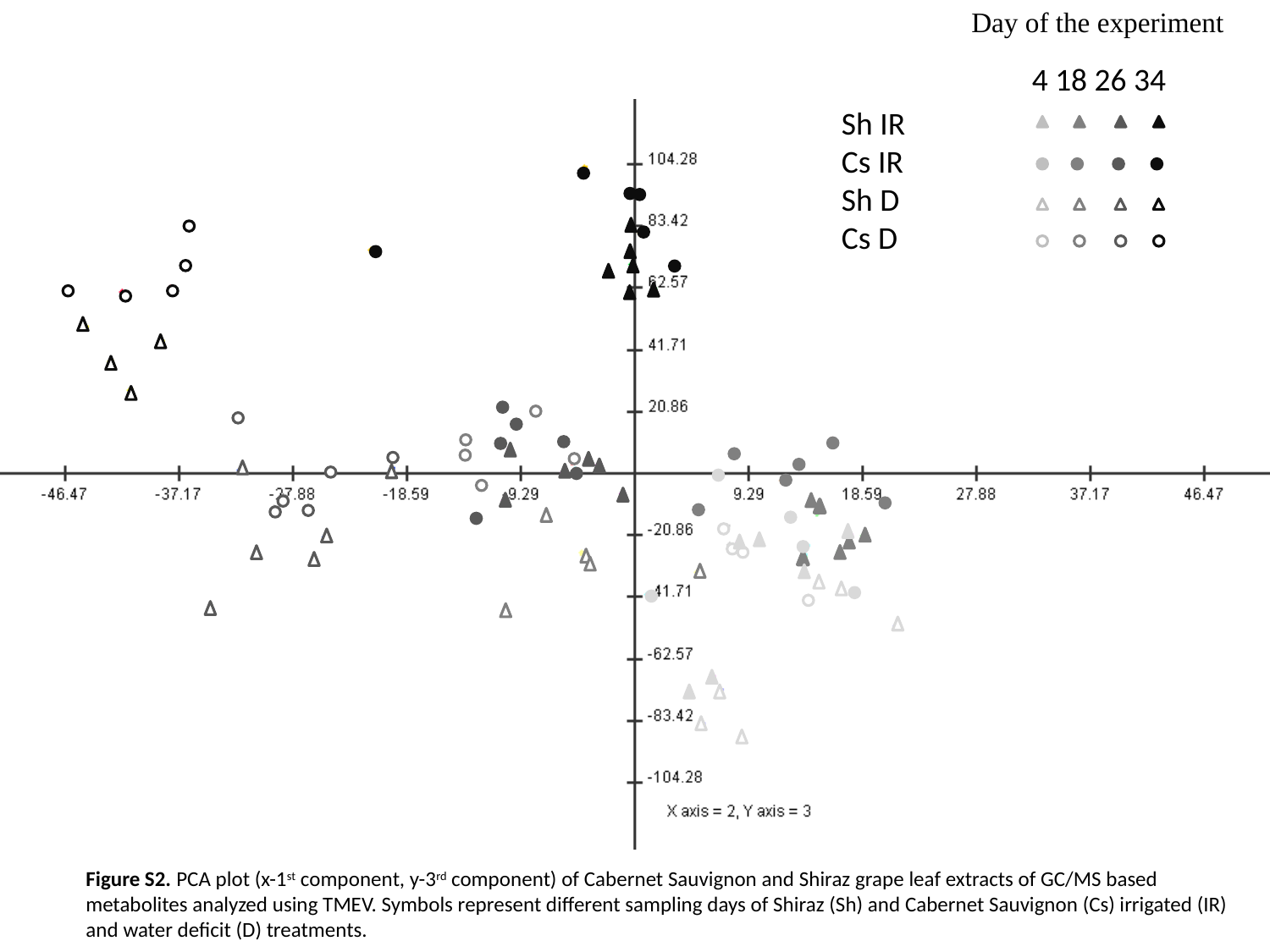

Day of the experiment
4 18 26 34
Sh IR
Cs IR
Sh D
Cs D
Figure S2. PCA plot (x-1st component, y-3rd component) of Cabernet Sauvignon and Shiraz grape leaf extracts of GC/MS based metabolites analyzed using TMEV. Symbols represent different sampling days of Shiraz (Sh) and Cabernet Sauvignon (Cs) irrigated (IR) and water deficit (D) treatments.

## Slide 3
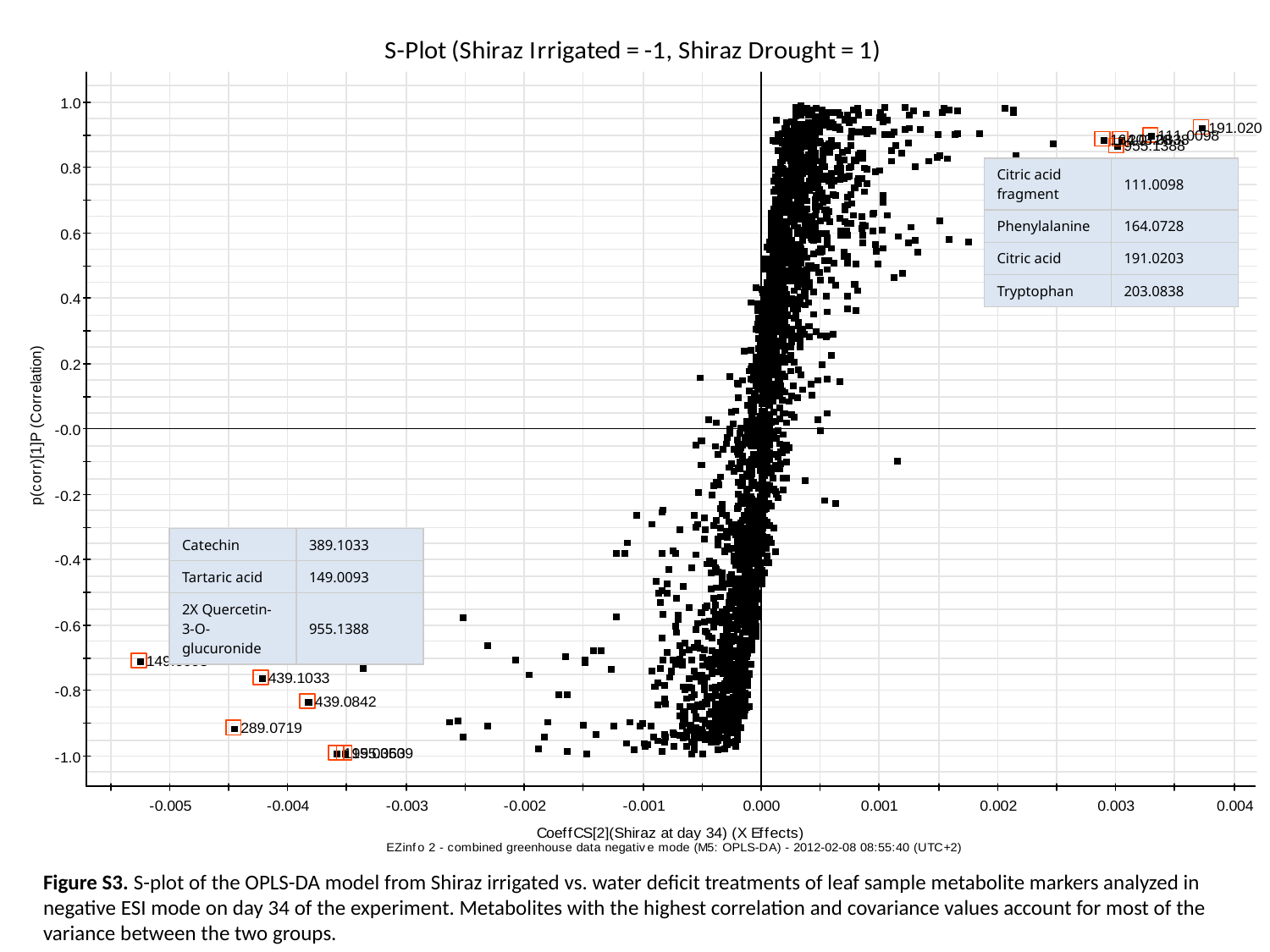

| Citric acid fragment | 111.0098 |
| --- | --- |
| Phenylalanine | 164.0728 |
| Citric acid | 191.0203 |
| Tryptophan | 203.0838 |
| Catechin | 389.1033 |
| --- | --- |
| Tartaric acid | 149.0093 |
| 2X Quercetin-3-O-glucuronide | 955.1388 |
Figure S3. S-plot of the OPLS-DA model from Shiraz irrigated vs. water deficit treatments of leaf sample metabolite markers analyzed in negative ESI mode on day 34 of the experiment. Metabolites with the highest correlation and covariance values account for most of the variance between the two groups.

## Slide 4
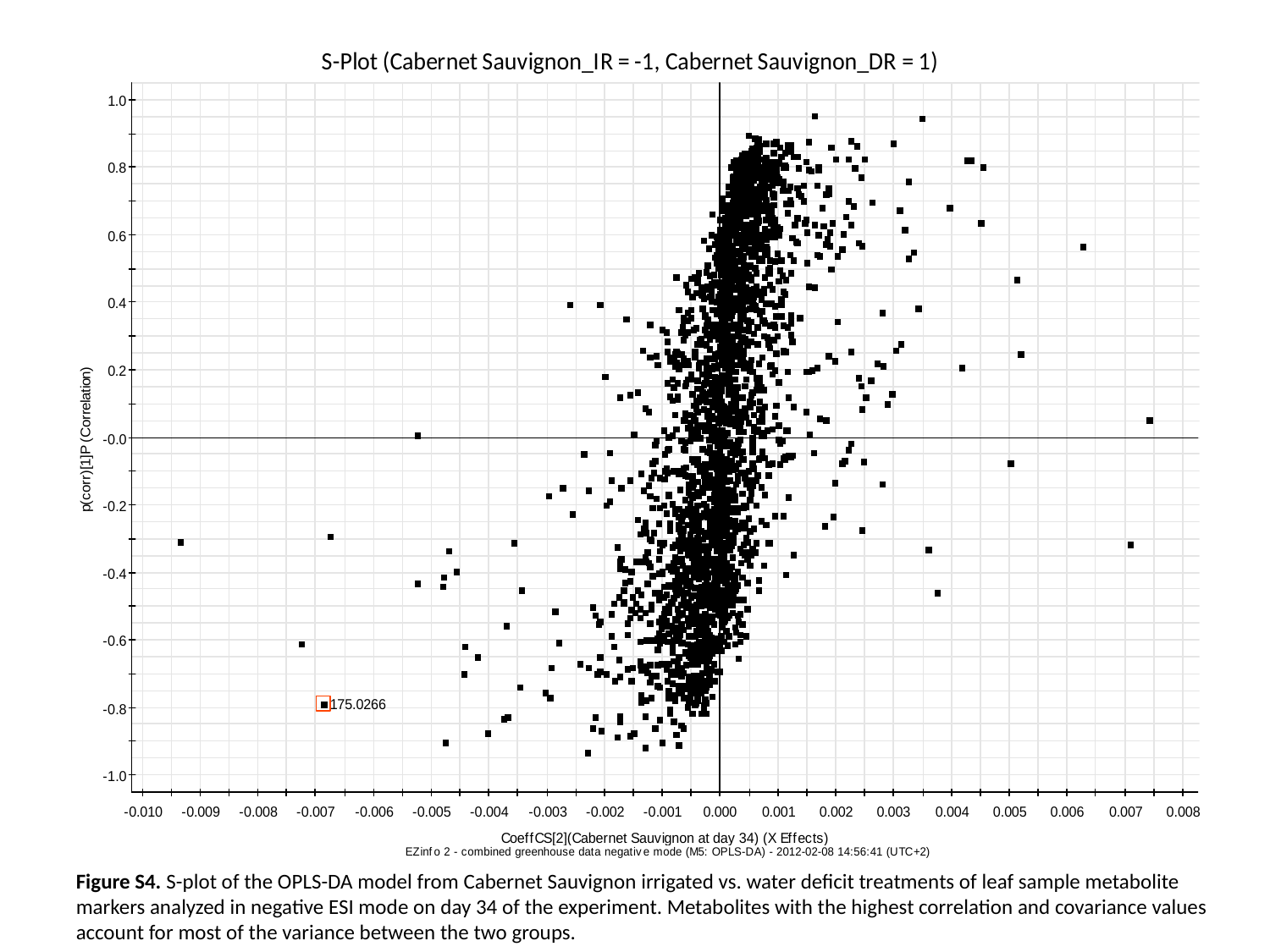

#
Figure S4. S-plot of the OPLS-DA model from Cabernet Sauvignon irrigated vs. water deficit treatments of leaf sample metabolite markers analyzed in negative ESI mode on day 34 of the experiment. Metabolites with the highest correlation and covariance values account for most of the variance between the two groups.

## Slide 5
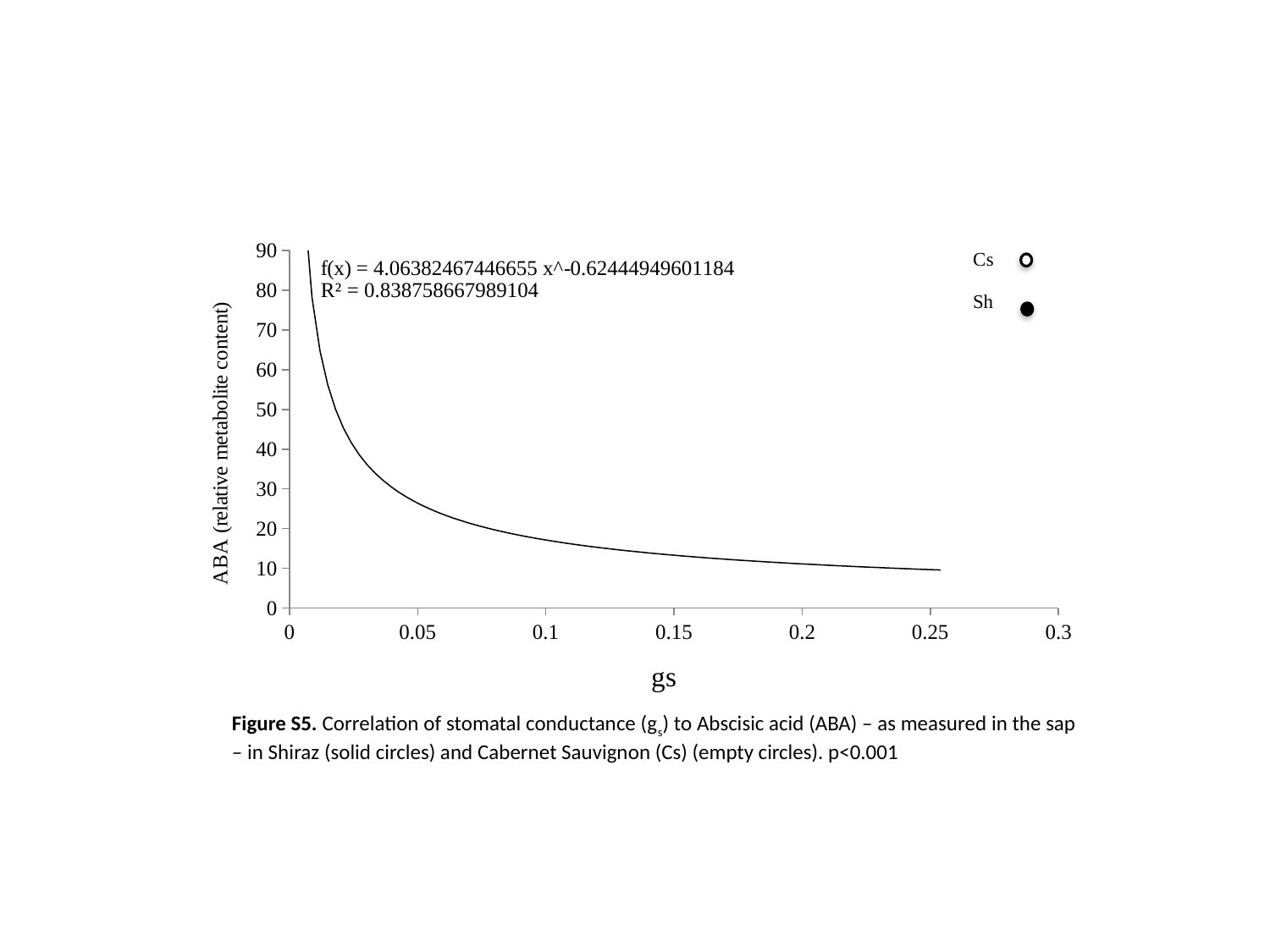

### Chart
| Category | |
|---|---|Figure S5. Correlation of stomatal conductance (gs) to Abscisic acid (ABA) – as measured in the sap – in Shiraz (solid circles) and Cabernet Sauvignon (Cs) (empty circles). p<0.001

## Slide 6
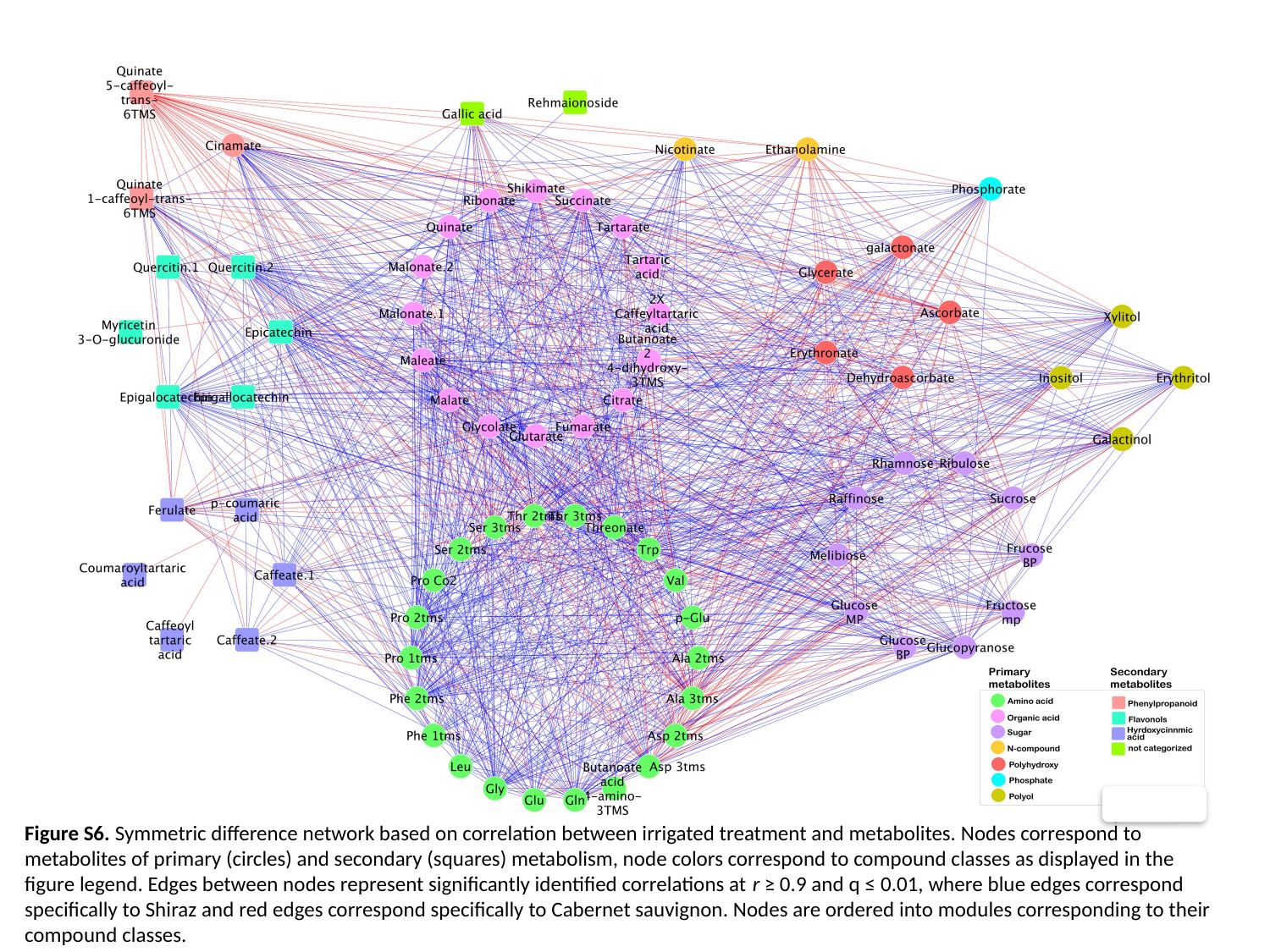

#
Figure S6. Symmetric difference network based on correlation between irrigated treatment and metabolites. Nodes correspond to metabolites of primary (circles) and secondary (squares) metabolism, node colors correspond to compound classes as displayed in the figure legend. Edges between nodes represent significantly identified correlations at r ≥ 0.9 and q ≤ 0.01, where blue edges correspond specifically to Shiraz and red edges correspond specifically to Cabernet sauvignon. Nodes are ordered into modules corresponding to their compound classes.

## Slide 7
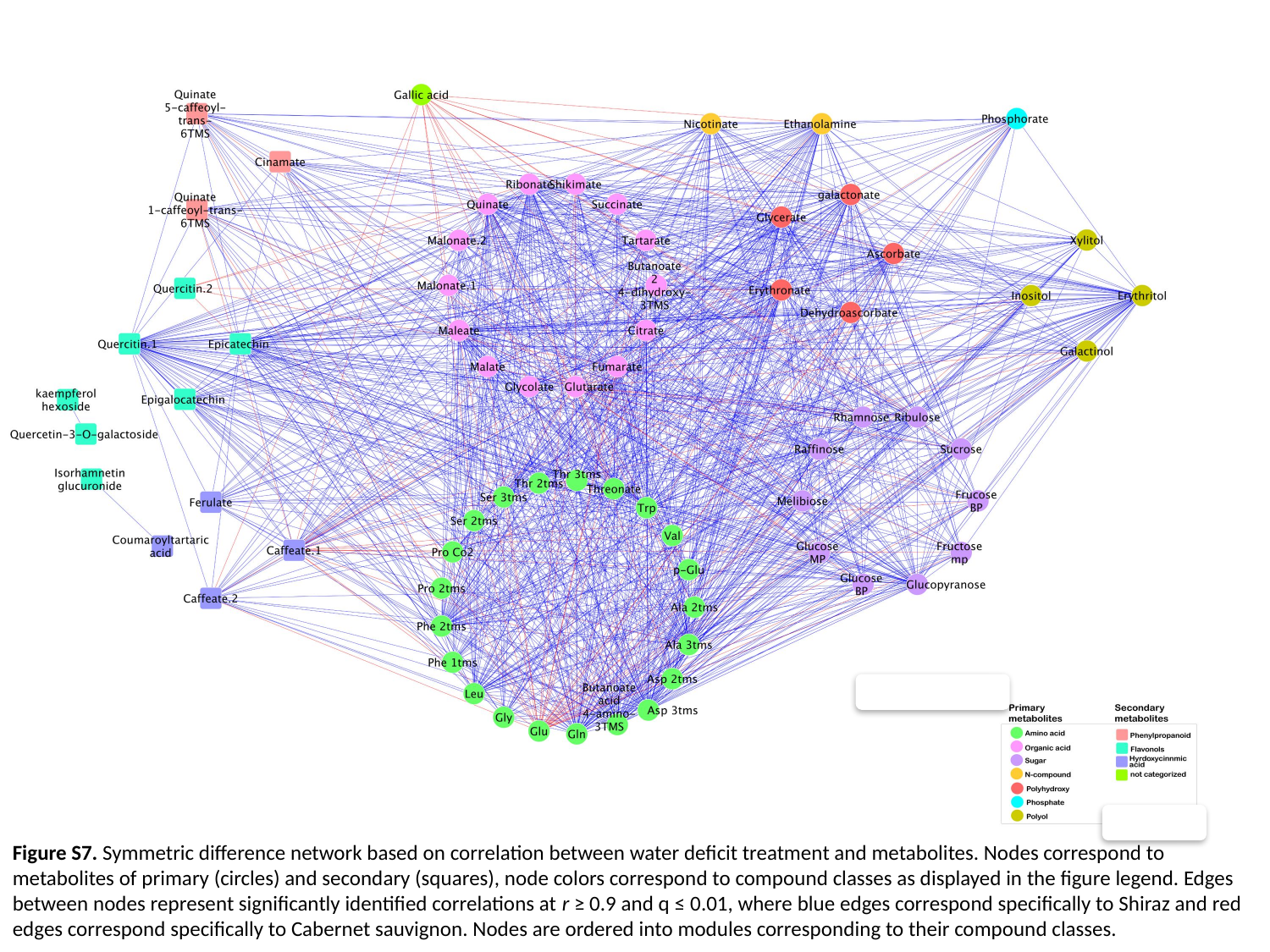

#
Figure S7. Symmetric difference network based on correlation between water deficit treatment and metabolites. Nodes correspond to metabolites of primary (circles) and secondary (squares), node colors correspond to compound classes as displayed in the figure legend. Edges between nodes represent significantly identified correlations at r ≥ 0.9 and q ≤ 0.01, where blue edges correspond specifically to Shiraz and red edges correspond specifically to Cabernet sauvignon. Nodes are ordered into modules corresponding to their compound classes.
